# Functional characterization of cutinase genes NsCut1-NsCut4 in *Neostagonosporella sichuanensis* and their effects on fishscale bamboo

**DOI:** 10.3389/fpls.2025.1564651

**Published:** 2025-04-08

**Authors:** Mengyao Lu, Fang Liang, Lijuan Liu, Yanji Yin, Dongxin Xu, Huan Zou, Yinggao Liu, Chunlin Yang

**Affiliations:** ^1^ College of Forestry, Sichuan Agricultural University, Chengdu, China; ^2^ National Forestry and Grassland Administration Key Laboratory of Forest Resources Conservation and Ecological Safety on the Upper Reaches of the Yangtze River, College of Forestry, Sichuan Agricultural University, Chengdu, China

**Keywords:** *Neostagonosporella sichuanensis*, cutinase, enzymatic characterization, gene knockout, pathogenicity

## Abstract

Fishscale bamboo rhombic-spot, caused by *Neostagonosporella sichuanensis*, poses a significant threat to *Phyllostachys heteroclada* in Sichuan province. Based on genomic analysis, four cutinase genes, NsCut1–NsCut4, were identified, cloned, and functionally validated. Bioinformatics analyses revealed that the proteins encoded by these genes possess secretory functions, lack transmembrane domains, and contain conserved cutinase domains highly homologous to those in other fungi. Recombinant proteins expressed via a prokaryotic system exhibited strong hydrolytic activity against glycerol tributyrate and bamboo white cream at 40°C and pH 8.0, while signal peptide and subcellular localization analyses confirmed their secretory function and localization to the cell wall. Gene knockout experiments were performed to construct deletion strains *ΔNsCut* and corresponding complemented strains *ΔNsCut+*. Notably, *ΔNsCut1* and *ΔNsCut3* resulted in reduced pigmentation, decreased spore production, and increased sensitivity to NaCl, H_2_O_2_, and Congo red, along with reduced pathogenicity—indicating that these genes play key roles in metabolic and reproductive processes, oxidative stress responses, and the maintenance of cell wall integrity. In contrast, *ΔNsCut2* and *ΔNsCut4* did not exhibit significant differences compared to the wild type. This work advances our understanding of the role of cutinases in the pathogenic interaction between *N. sichuanensis* and *P. heteroclada*, providing a theoretical basis for further exploration of the pathogen’s underlying mechanisms.

## 1 Introduction

*Phyllostachys heteroclada* (fishscale bamboo) is a common bamboo species in Southwest China, serving as food, medicine, and a significant bamboo timber crop (Lin, 2005; Yang et al., 2019; Dai et al., 2023). It is crucial in promoting high-quality bamboo industry growth and maintaining regional ecological balance. However, in recent years, rhombic spot disease has become widespread in *P. heteroclada* forest, causing death and deterioration. *Neostagonosporella sichuanensis* is the primary pathogen for this disease (Yang, 2019; Yang et al., 2024). It primarily affects mature bamboo (older than 0.5 years), causing rhombic spots on the culm and leading to the withering of tips, branches, and, eventually, the entire plant. Severe infestations can weaken or even kill *P. heteroclada* within 1 to 3 years.

In recent years, significant progress has been made in the genomic characterization, pathogenesis, and ecological adaptation of *N. sichuanensis*. Genome-level studies revealed that the pathogen has a remarkable bipartite structure, with a concentrated distribution of pathogenicity-related genes, especially those related to plant cell wall degradation, secondary metabolism regulation, and signaling, whose functions significantly enhance the competitiveness of the pathogen for survival in complex environments (Liu et al., 2024b). It was found that *Nsxyn1* and *Nsxyn2* of the GH11 family of xylanase genes exhibited efficient xylanase activity and were able to efficiently decompose xylan components in plant cell walls to promote pathogen infection (Liu et al., 2024a). Whereas *NsCFEM1*, one of the proteins in the CFEM structural domain of *N. sichuanensis*, inhibited Bax-induced programmed cell death of Benthic tobacco cells, suggesting that it acted as a virulence factor, *NsCFEM2* contributed to the stability of the cell wall (Liang et al., 2024). In addition, studies on the interfollicular fungal community of *P. heteroclada* showed that the pathogen might dominate the community through a competitive exclusion mechanism. At the same time, interactions with other commensal fungi enhanced their ecological adaptability (Liu et al., 2022). These findings provide new perspectives for the revelation of the pathogenic mechanism of *N. sichuanensis* and the development of disease prevention and control strategies.

Most plant diseases damage the cuticle to facilitate infection by generating cutinase (Skamnioti and Gurr, 2007; Fu et al., 2020; Dong et al., 2021). Cutinase is a serine esterase classified as a member of the α/β hydrolase superfamily that breaks down cutin polyesters (Chen et al., 2013). Fungal cutinase genes often comprise two disulfide bonds and a phosphorylated protein kinase site (Lin and Kolattukudy, 1980). Most cutinases contain the conserved GYSQG structural domain and the similar DXVCXG[ST]-[LIVMF](3)-X(3)H motif, which play a vital role in the function and stability of cutinases (Longhi et al., 1997; Chen et al., 2013). They have the classical Ser-His-ASP catalytic structure, which grants them the capability to break down various substances, such as slightly soluble esters and both short- and long-chain triacylglycerols (Longhi and Cambillau, 1999; Gong et al., 2022). Although cutinases exhibit similarities in conserved structural domains and amino acid sequences, they vary significantly regarding molecular weight, temperature adaptability, pH preference, substrate specificity, and stability among various organisms (Nikolaivits et al., 2018).

The cuticle, a biopolyester in the outermost layer of higher plants, mainly consists of hydroxy fatty acids with sixteen and eighteen carbon atoms and epoxy fatty acids (Nawrath, 2006; Li, 2008). This composition gives the cuticle hydrophobic properties, making it the first plant defense line against external stressors (Chen et al., 2013; Villafana and Rampersad, 2020). Many studies have shown that the cuticle acts as a physical barrier and is involved in signal generation and transmission during plant growth, development, and interaction with pathogens (Bird and Gray, 2003; Skamnioti and Gurr, 2007; Li, 2008; Villafana and Rampersad, 2020). Cuticle structure and permeability changes can affect the plant’s ability to defend against pathogenic fungi (Chassot et al., 2007; Arya et al., 2021). It has been found that CAZymes are a family of proteins with various glycocatalytic activities, which are usually closely related to fungal growth and development and pathogenicity (Zhou et al., 2021). Cutinases usually belong to the CE5 family of CAZymes (Chen et al., 2013). Heat map clustering analysis of the CE5 family of *N. sichuanensis* with published genomic resources and 17 other different trophic fungi (including dead trophic fungi, semi-living trophic fungi, living trophic fungi, saprophytic fungi, commensal fungi, fungal parasites, and epiphytic fungi) from the same family showed that compared with the non-pathogenic fungi and the living trophic fungi, the CE5 family was expanded in *N. sichuanensis* genome (Liu et al., 2024b). Expansion and contraction of a gene family in a fungus may be related to its adaptation to the host plant, suggesting that the cutinase gene of *N. sichuanensis* may play a role in the infestation of the host by the pathogen.

Cutinase can degrade cutin polymers in plant cell walls, aiding pathogen invasion of host cells (Dong et al., 2021; Gong et al., 2022). Deletion mutants of two cutinase genes in *Arthrinium phaeospermum* had lower disease indices on hybrid bamboos than the wild-type (Fang et al., 2021; Yan et al., 2022). Inserting *Fusarium solani* f. sp. *pisi* cutinase gene into *Mycosphaerella* spp. led to infestation of intact papayas (Stahl and Schfer, 1992; Stahl et al., 1994). *Alternaria longipes* shortens pathogenic incubation and exacerbates disease by adding purified cutinase to conidial suspensions and inoculating tobacco leaves (Liu, 2009). Cutinases enhance fungal spore adhesion to plants and promote pathogen infection (Liang et al., 2003). For example, *Uromyces fabae* and *Colletotrichum graminicola* cutinases are involved in pathogen attachment (Köller, 1991; Francis et al., 1996). Some pathogen cutinase gene knockouts do not affect virulence (e.g., *Botrytis cinerea*, *Fusarium graminearum*, *Nectria haematococca*, *Ustilaginoidea virens*) (Stahl and Schfer, 1992; Crowhurst et al., 1997; van Kan et al., 1997; Chen et al., 2021). Cutinases are involved in signal transduction and plant immune responses in host-pathogen interactions (Soliday and Kolattukudy, 1983; Bessire et al., 2007). For instance, *Rhizoctonia zeae* cutinase protein *RCCUT1* induces plant cell necrosis (Lu et al., 2018). Similarly, *A. phaeospermum* cutinase genes can inhibit host immune responses (Fang et al., 2021). Findings highlight the pivotal role of cutinases in mediating host-pathogen interactions.

In this study, we successfully cloned and characterized the functions of four cutinase genes from *Neostagonosporella sichuanensis*. This work advances our understanding of the role of cutinases in the pathogenic interaction between *N. sichuanensis* and *Phyllostachys heteroclada*, providing a theoretical basis for further exploration of the pathogen’s underlying mechanisms.

## 2 Materials and methods

### 2.1 Plant, strains and culture conditions

One-year-old *Phyllostachys heteroclada* was transplanted from Ya’an to an experimental greenhouse on the Chengdu Campus of Sichuan Agricultural University. *Neostagonosporella sichuanensis* wild-type strain (SICAUCC 16-0001) was obtained from diseased branches of *P. heteroclada* in Ya’an City, Sichuan Province, China, inoculated on PDA agar medium and then incubated at 25°C for 30 d. The knockout and complementary strains of NsCut1-NsCut4 were cultured on a PDA agar medium containing hygromycin B or Geneticin (G418) at 25°C.

*Escherichia coli* DH5α was cultured on LB agar medium containing ampicillin (Amp) at 37°C. Positive DH5α colonies were cultured in liquid LB agar medium containing Amp at 37°C with 220 r min^-1^ oscillation. *Agrobacterium tumefaciens* GV3101 (pSoup-19), and its positive colonies were similarly cultured in LB agar medium containing 20 μg mL^-1^ rifampicin and 20 μg mL^-1^ kanamycin at 28°C. In addition, *Saccharomyces cerevisiae* YTK12 strain was cultured on yeast extract PDA agar medium (YPDA) at 30°C and positive colonies were cultured on CMD-W agar medium (6.7 g Yeast nitrogen base without amino acid, 0.75 g tryptophan dropout supplement, 20 g sucrose, 1 g glucose, 20 g agar) and YPRAA agar medium (10 g yeast paste, 20 g peptone, 20 g raffinose, 20 g agar) at 30°C.

### 2.2 Identification and bioinformatics analysis of the cutinase family of *Neostagonosporella sichuanensis*

The genes containing the Cutinase structural domain (Pfam ID: PF01083) were screened in the whole genome of *Neostagonosporella sichuanensis* using the platform provided by the sequencing company. The screening results were validated with HMMER v3.4 (E-value threshold ≤ 1×10^-5^) to ensure the reliability of the structural domain prediction. Upon identification, a total of four cutinase genes were screened, which were named *NsCut1*, *NsCut2*, *NsCut3*, and *NsCut4*. Open reading frame analysis was performed with DNAMAN software to determine the gene lengths of the NsCut1-NsCut4, and physicochemical properties were calculated using the standard algorithm provided by Expasy ProtParam (https://web.expasy.org/protparam/).

Transmembrane structural domains were identified using TMHMM-2.0 (https://services.healthtech.dtu.dk/service.php?TMHMM-2.0), glycosylation sites were analyzed with NetGlycate-1.0 (https://services.healthtech.dtu.dk/services/NetGlycate-1.0/), and signal peptides were predicted using SignalP-5.0 (https://services.healthtech.dtu.dk/service.php?SignalP-5.0), while subcellular localization was predicted using Euk-mPLoc 2.0 (http://www.csbio.sjtu.edu.cn/bioinf/euk-multi-2/). Phylogenetic analysis was carried out using MEGA v11 to construct an evolutionary tree by the Neighbor-joining method (Tamura et al., 2021). Multiple sequence alignment was performed using ESPript 3.0 (https://espript.ibcp.fr/ESPript/cgi-bin/ESPript.cgi). The secondary and tertiary structures of the four cutinases were predicted using SOMPA (https://npsa.lyon.inserm.fr/cgi-bin/npsa_automat.pl?page=/NPSA/npsa_sopma.html) and AlphaFold3 (https://alphafoldserver.com/), respectively, and the model quality was assessed by PROCHECK, QMEAN, and triple validation through model overlay comparison using UCSF Chimera (Laskowski et al., 1996; Pettersen et al., 2004; Benkert et al., 2009; Abramson et al., 2024).

### 2.3 Cloning of the NsCut1-NsCut4 genes

The CDS region of the cutinase gene from *Neostagonosporella sichuanensis* was used as a template, and Primer Premier 5.0 software was employed to design gene-specific cloning primers. (**Supplementary Figure 1**). Total RNA was extracted from fresh mycelium, and its quality was assessed by electrophoresis and spectrophotometry, followed by reverse transcription to synthesize cDNA (Zhao et al., 2022). The target genes were amplified by PCR using high-fidelity enzymes with cDNA as the template, and the amplified products were detected by electrophoresis. The target gene fragments were recovered and ligated with the pMD19T vector to construct the recombinant vector pMD19T-(NsCut1-NsCut4). The recombinant plasmid was transformed into *Escherichia coli* DH5α, positive clones were screened and then identified by colony PCR, and the successfully transformed fungal fluids were sequenced to obtain the correct recombinant plasmid, which was finally stored at -80°C.

### 2.4 Construction of prokaryotic expression system and purification of recombinant protein

The recombinant plasmid pMD19T-(NsCut1-NsCut4) was extracted using the High Purity Plasmid Extraction Kit. The prokaryotic expression vector pET32a-(NsCut1-NsCut4) was constructed by *EcoR*I and *Xho*I digestion and transformed into *Escherichia coli* BL21 (DE3) for expression (**Supplementary Figure 2**). IPTG was used to induce recombinant protein expression, and the induction effect was detected by SDS-PAGE after incubation at 37°C for 3 h. Protein purification was performed using the His-tag Protein Purification Kit from Beyotime with protein concentration determined using the Bradford Protein Concentration Assay Kit from Solarbio. The concentration of purified recombinant protein was calculated and recorded.

### 2.5 Recombinant cutinase activity assay

Several methods were used to determine the enzymatic activity of cutinase from *Neostagonosporella sichuanensis*. The hyaline circle method was used to detect the enzyme activity of purified proteins in glycerol tributyrate agar medium (0.5% glycerol tributyrate, 0.05 M PBS (pH=7.0), 1% agar) and to observe the formation of hyaline circles (Kwon et al., 2009). The modified Zapek-Dox medium method utilized *Phyllachachys heterocluanada* bamboo white cream as the only carbon source, and the ability of cutin degradation was assessed by the colour change of the indicator cresol red (purplish-red to yellow) (Dickman and Patil, 1986). The p-NPB method used p-nitrophenyl butyrate as a substrate to determine the enzyme activity under specific reaction conditions, defining the production of 1 μg of p-nitrophenol per minute as one unit of enzyme activity (1 U mL^-1^) (Lv et al., 2022).


$$\text{U}=\frac{OD\times \text{V}\times \text{Mr}}{\text{ϵ}\times \text{λ}\times \text{V}1\times \text{T}}$$

Where U is the cutinase activity; OD is the absorbance value; V is the total volume of the reaction system (1.8 mL); Mr is the molecular weight of p-nitrophenol (139.11 g mol^-1^); ϵ is the molar extinction coefficient (6830 L mol^-1^cm^-1^); λ is the optical range of the cuvette (0.5 cm); V1 is the volume of enzyme liquid (200 μL); T is the reaction time (10 min).

### 2.6 Enzymatic characterization of recombinant cutinases

In this study, the optimum reaction conditions and stability of recombinant cutinase were systematically analysed. The optimum reaction temperature was determined by measuring the enzyme activity using p-NPB as a substrate at different temperatures (20-70°C). The residual enzyme activity of the enzyme solution was assessed by a thermal stability test after incubating the enzyme solution at different temperatures for 1 h.

BR buffer at pH 3-10 was prepared using 20 mmol L^-1^ NaOH to assess the optimal pH and stability of recombinant cutinase under different pH conditions.

The effect of metal ions on enzyme activity was investigated by adding Ca^2+^, Zn^2+^, Co^2+^, Cu^2+^, Mg^2+^, Fe^2+^, Ba^+^, Mn^2+^, Cr^+^, K^+^, and Na^+^. Their effects on enzyme activity were determined at 5 mmol L^-1^ and 10 mmol L^-1^ concentrations.

Additionally, the effect of different surfactants and inhibitors (Tween 20, Tween 80, SDS, EDTA, and Triton X-100) on cutinase activity was investigated to determine the inhibitory effect of these reagents on enzyme activity. All experiments were repeated three times, and the results were expressed as relative enzyme activity.

### 2.7 The yeast secretion trap screen assay

The recombinant plasmid pSUC2-(NsCut1-NsCut4)^sp^ was constructed by linearising the pSUC2 vector using *EcoR*I and *Xho*I restriction endonucleases in combination with the target gene fragment containing the homology arms. Positive colonies were validated by the primer pSUC2-yz-F/R after being transformed into DH5α receptor cells (**Supplementary Figure 3**). Signal peptide secretion function was verified by the yeast signal peptide screening system (Qi et al., 2020; Wang, 2020). Pichia yeast YTK12 receptor cells were prepared, and the recombinant plasmid was transformed into yeast using PEG/LiAc-mediated heat-excited transformation. The transformed strain was cultured on CMD-W medium to screen for positive colonies. The positive colonies were transferred to YPRAA agar medium, and continued growth on this medium indicated that the signal peptide possessed secretion activity. Additionally, the signal peptide activity was further confirmed by TTC staining assay. Positive fungal fluid exhibited red precipitation in the TTC solution, demonstrating that the signal peptide was able to secrete sucrose-converting enzyme to the outside of the cell and functioned effectively, thereby confirming that the signal peptide of the target gene had secretion capability (Li, 2022).

### 2.8 Subcellular localization

The subcellular localization of NsCut1-NsCut4 was conducted using Agrobacterium-mediated transient expression in onion epidermal cells (Gong et al., 2020). The PCAMBIA super1300-GFP vector was linearised by *Xba*I and *Kpn*I cleavage to obtain a target gene fragment with the stop codon removed and with homologous arm sequences. The recombinant plasmid GFP-(NsCut1-NsCut4) was constructed and transformed into DH5α cells (**Supplementary Figure 4**). The recombinant plasmid was transformed into *Agrobacterium tumefaciens* GV3101(pSoup-19) competent cells, which were then coated on LB agar medium and cultured at 28°C for 48 h. Positive colonies were selected for expansion. The bacterial cultures were collected by centrifugation, washed three times with injection buffer, resuspended, adjusted to an OD600 of 0.1, and left in darkness at room temperature for 2 h. The pre-cultured onion inner epidermis was immersed in Agrobacterium suspension for 20 min, then spread on MS agar medium and cultured at 25°C under alternating light-dark for 24-48 h. Green fluorescent protein expression was observed under a fluorescence microimaging system. The tissue was infiltrated with 30% sucrose solution for 5 min to observe green fluorescence distribution after plasmodesmata separation.

### 2.9 Construction and identification of knockout mutants

For the construction of knockout vectors, DNA was extracted from *Neostagonosporella sichuanensis* (SICAUCC 16-0001), and the CDS region of the cutinase genes NsCut1-NsCut4 was selected to amplify the upstream and downstream homology arms, each spanning 1100-1500 bp. The Hygromycin B resistance gene (*hph*) was also amplified (**Supplementary Figure 5**). Homologous recombination of these fragments with a linearised pCE-Zero vector was performed to construct the knockout vector pCE-Zero-upstream homology arm-*hph*-downstream homology arm. The constructed vector was transformed into *Escherichia coli* DH5α, and positive clones were identified by PCR and sequencing. Plasmids were extracted and stored for backup.

In the protoplast preparation process, wild-type strains were first cultured and the mycelium was enzymatically digested using an enzymatic solution. After digestion, protoplasts were purified and counted. Knockout vectors were introduced into the protoplasts by PEG-mediated gene transformation, and positive transformants were screened on medium containing Hygromycin B and G418. The knockout of the target gene was confirmed by PCR validation (**Supplementary Figure 6**).

### 2.10 Functional backfilling of knockout mutants

For functional backfilling, DNA was extracted from wild-type strains to amplify the NsCut1-NsCut4 genes and their promoter sequences, which were then cloned into the pEASY-NeoR vector. The vector was linearised by *Hind*III digestion, and homologous recombination with the target gene fragments was performed to construct the complementation vector. The recombinant vector was transformed into *Escherichia coli* DH5α for sequencing verification. The validated plasmids were transformed into the corresponding knockout mutants, and the complementation transformants were screened by G418. PCR was then employed to detect the target genes in the cDNA to confirm the successful complementation. The successfully complementary strain was named Δ*NsCut+*.

### 2.11 Phenotypic analysis and stress tolerance assay of knockout and complementary strains

To analyze the differences in nutrient growth among wild-type, knockout and complementary strains, each strain was inoculated separately onto PDA plates and incubated at 25°C under alternating light and dark cycles for 12 h. The mycelial growth diameter was measured daily using the crossover method, and growth was observed. Three replicates were performed for each strain, and the experiment was repeated three times.

A previous study demonstrated that wild-type strains of *Neostagonosporella sichuanensis* could be cultured indoors, but spore production was not detected (Qi et al., 2021). Subsequently, we identified that lactose agar medium supplemented with *Phyllostachys heteroclada* culm broth could induce spore production in *N. sichuanensis*. Therefore, each strain was inoculated on bamboo culm decoction agar medium at 25°C for 30 d, after which spore production was observed. The mycelium was washed with 1 mL ddH_2_O, and the conidia were collected. The spore morphology was observed under a microscope and the spore production was counted using a hemocytometer plate. The spore suspension was diluted to 1×10^4^ mL^-1^, and 20 μL of the spore solution was applied dropwise onto a slide. Spore germination was observed after 24 h of humidified incubation.

Using modified Zapek-Dox selective medium, each strain was inoculated and incubated at 25°C for 30 d. Cutinase secretion was assessed based on the color change of the indicator (purplish-red to yellow), and the area of color change was measured to evaluate differences in secretion levels.

Stress tolerance assays were conducted using PDA medium to assess the differences in stress responses among the strains. Wild-type, knockout, and complementary strains were inoculated onto PDA medium containing 2 mol L^-1^ NaCl, 2 mg L^-1^ Congo red, and 40 mmol L^-1^ H_2_O_2_, respectively. Mycelial growth was monitored, and the mycelial diameter was measured to calculate the relative inhibition rate. Three replicates were set for each condition.

### 2.12 Pathogenicity testing of knockout and complementary strains

One-year-old healthy *Phyllostachys heteroclada* with a consistent genetic background were randomly selected for pathogenicity testing. Conidia from each strain were collected and transferred into a spore suspension containing 10 mL of sterile distilled water. Prior to inoculation, the surfaces of bamboo stems were rinsed with sterile water and gently wiped with a degreasing cotton ball moistened with 70% ethanol for surface disinfection. The bamboo stems were then pricked with a sterile insect needle, and the spore suspension was applied to the surface of the bamboo stems using a sterilized brush. The samples were bagged, moisturized, and treated every 12 hours (9 am and 9 pm). A control group was treated with sterile water. Conventional cultivation and management methods were employed to observe the symptoms of the plants, and disease surveys were done for the onset of the disease at 7 d, 14 d, 21 d, 28 d, 35 d, and 42 d post-inoculation. The data were analyzed by one-way ANOVA and Duncan’s test using SPSS 16.0 software to analyze the significance of differences in disease indices.

## 3 Results

### 3.1 Analysis of amino acid sequence and protein structure

Using DNAMAN sequence analysis software, we determined that the gene lengths of *NsCut1*, *NsCut2*, *NsCut3*, and *NsCut4* are 687 bp, 735 bp, 672 bp, and 942 bp, respectively (**Supplementary Figure 1**). Physicochemical property analyses revealed that *NsCut1* and *NsCut3* are hydrophilic proteins, whereas *NsCut2* and *NsCut4* are hydrophobic. In addition, no transmembrane structural domains were detected in any of the four proteins, and each protein contains at least one glycosylation site and a signal peptide, suggesting that they are secreted proteins (**Supplementary Figure 2**). Secondary structural analyses further showed that all four proteins possess a typical disulfide bond structure: specifically, *NsCut1* contains eight cysteine residues forming four disulfide bonds, whereas *NsCut2*, *NsCut3*, and *NsCut4* each contain four cysteine residues forming two disulfide bonds (**Supplementary Figure 3**). All four proteins also exhibit similar numbers and positions of α-helices, η-helices, β-folds, and strict β-folds (TTT).

### 3.2 Model quality assessment and structural overlay analysis

**Figure 1:**
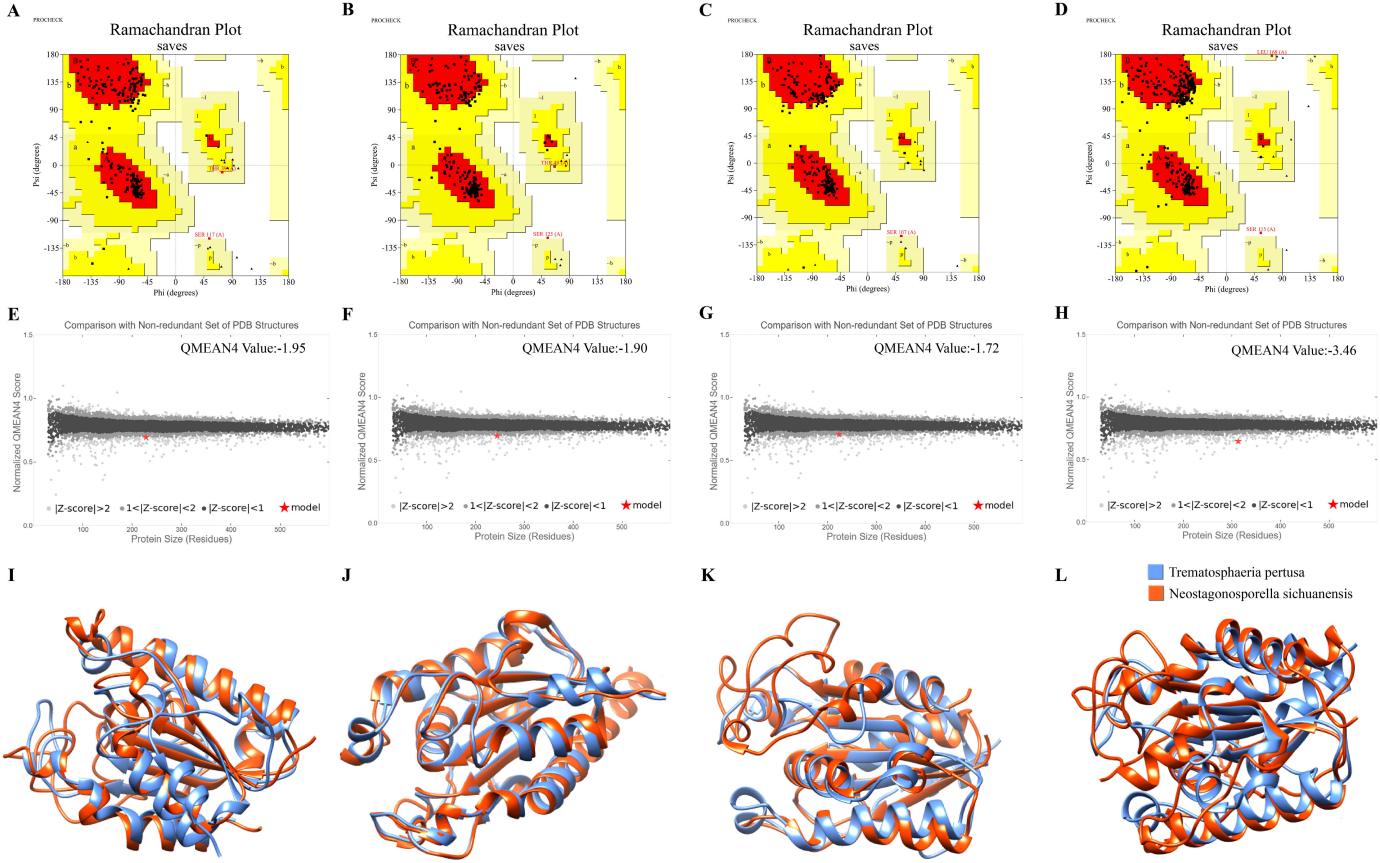
Model quality assessment and structural overlay analysis. **(A-D)** PROCHECK assessments for each of the four protein models NsCut1-NsCut4 predicted by AlphaFold3. **(E-H)** The four protein models of NsCut1-NsCut4 are separately used for the QMEAN assessment. **(I-L)** The structural overlay compared the four NsCut1-NsCut4 proteins’ tertiary structures with the Cutinase AlphaFold DB model of *Trematosphaeria pertusa*.

The tertiary structures of the four cutinase proteins were predicted using AlphaFold3 (**Supplementary Figure 3**). To assess the structural validity of NsCut1-NsCut4, we subjected these proteins to model quality assessment and structural overlay analysis with the Cutinase AlphaFold DB model of *Trematosphaeria pertusa*. PROCHECK analysis revealed that 86.7%, 92.7%, 91.0%, and 84.0% of the amino acid residues of *NsCut1*, *NsCut2*, *NsCut3*, and *NsCut4*, respectively, were located in the most favorable region, indicating high structural plausibility (**Figures 1A–D**). The Z-values of Qmean were -1.95, -1.90, -1.72, and -3.46, all of which fall within the acceptable range of |Z-score| < 4, further confirming the stability and reliability of the models (**Figures 1E–H**). Compared with the Cutinase AlphaFold DB model of *T. pertusa*, the tertiary structures of the four proteins showed low RMSD values (1.129 Å to 0.845 Å), suggesting a high degree of similarity between the structures and confirming that they belong to the same protein class (**Figures 1I–L**). In conclusion, the structural models of the four cutinase proteins are of high quality and are suitable for subsequent experimental analyses.

### 3.3 Functional domain analysis of NsCut1-NsCut4 proteins and their evolutionary relationship with cutinases of Phaeosphaeriaceae

**Figure 2:**
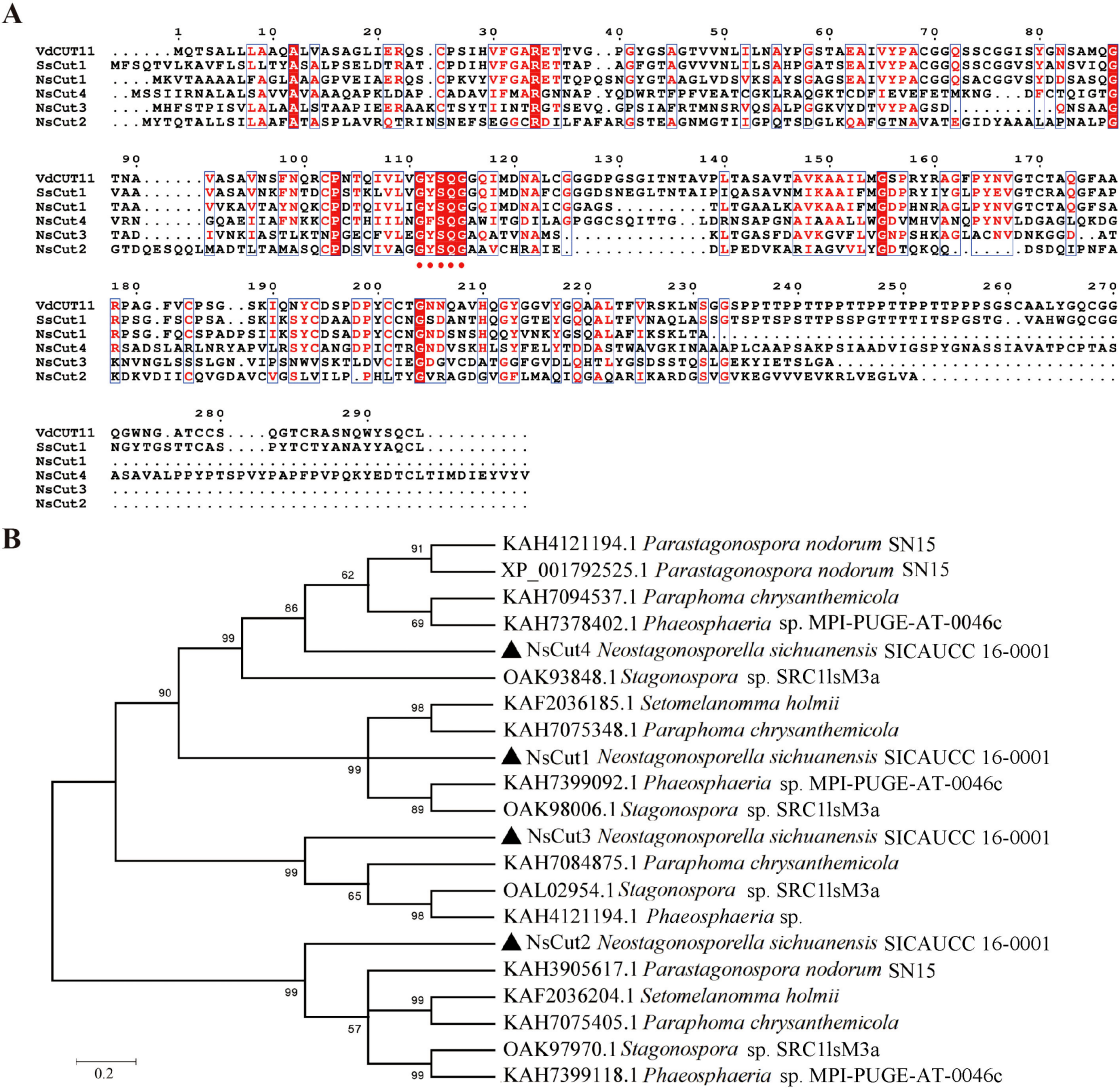
Multiple sequence alignment analysis and analysis of phylogenetic trees. **(A)** The reported protein sequences of the cutinase *VdCUT11* from *Verticillium dahliae* and the cutinase *SsCut1* from *Sclerotinia sclerotiorum* were used as reference sequences for homology analysis with NsCut1-NsCut4. Red dots indicate the conserved GYSQG catalytic site. **(B)** Evolutionary relationships of cutinase from the *Neostagonosporella sichuanensis* with other species of Phaeosphaeriaceae. NsCut1-NsCut4 are indicated as ▲.

Analyzed by NCBI prediction tool, NsCut1-NsCut4 proteins all contain conserved Cutinase structural domains and belong to the α/β hydrolase superfamily (**Figure 2A**). Homology analysis showed that *NsCut1*, *NsCut2*, and *NsCut3* have conserved GYSQG catalytic sites, while tyrosine (Y) was replaced by proline (F) in the catalytic site of *NsCut4*. Phylogenetic tree analyses showed that the cutinases of Phaeosphaeriaceae can be divided into four main branches. NsCut1-NsCut4 are each distributed on a distinct branch, similar to the cutinases of the other five species within Phaeosphaeriaceae (**Figure 2B**).

### 3.4 Successful cloning and purification of four cutinase

Four cutinase genes, NsCut1-NsCut4, were successfully cloned from the cDNA of *Neostagonosporella sichuanensis* (**Supplementary Figure 4**). We had planned to investigate the functions of these four cutinase proteins.

Recombinant plasmids pET32a-(NsCut1-NsCut4) were successfully constructed and subsequently transformed into *Escherichia coli* BL21 (DE3) cells (**Supplementary Figure 5**). To optimize the expression of NsCut1-NsCut4, the culture was expanded under the condition of IPTG final concentration of 0.2 mmol L^-1^ and induced for 3 h at 37°C. The recombinant proteins were purified in large quantities using a protein purification kit, resulting in four cutinase proteins with high purity (**Supplementary Figure 6**).

### 3.5 Purified recombinant protein has cutinase activity

**Figure 3:**
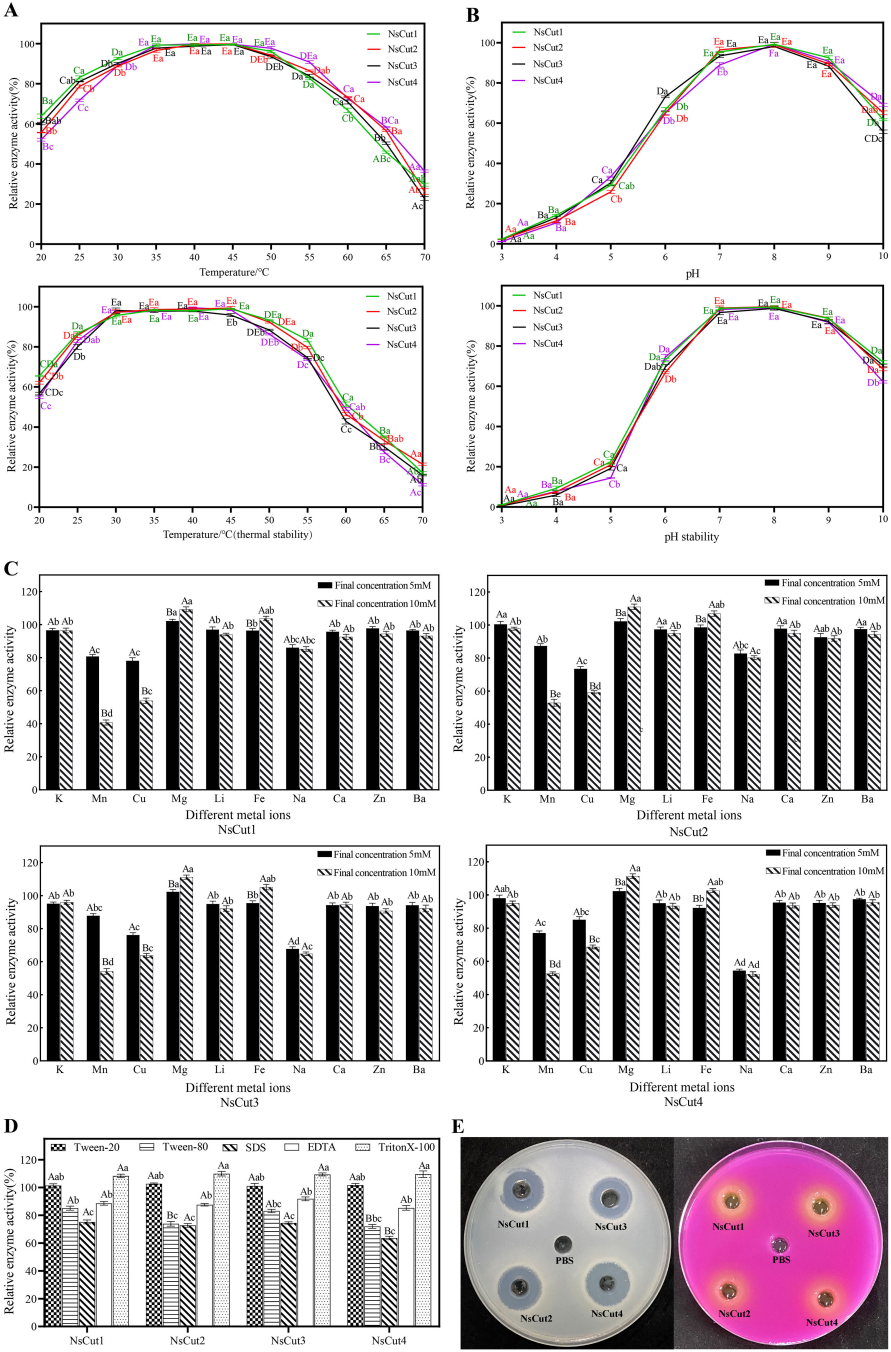
Effects of different temperatures, pH, metal ions, surfactants, and inhibitors on four cutinases and identification of enzyme activities. **(A)** Analysis of optimal temperature and thermal stability of recombinant proteins *NsCut1* to *NsCut4.* **(B)** Optimal pH and pH stability analysis of recombinant proteins *NsCut1* to *NsCut4*. Different uppercase letters are used to label significant differences in the relative enzyme activities of the same cutinase at different temperatures (pH) conditions; different lowercase letters are used to label significant differences in the relative enzyme activities of different cutinases at the same temperatures (pH) conditions (*P ≤* 0.05). **(C)** Effect of metal ions on recombinant proteins *NsCut1* to *NsCut4*. **(D)** Effect of surfactants and inhibitors on recombinant proteins *NsCut1* to *NsCut4*. Different uppercase letters are used to label significant differences in the relative enzyme activities of different cutinase under the same metal ion (surfactant and inhibitor) conditions; different lowercase letters are used to label significant differences in the relative enzyme activities of the same cutinase under different metal ion (surfactant and inhibitor) conditions (*P ≤* 0.05). **(E)** The left panel produces precise circles on the cutinase-degraded glycerol tributyrate medium, and the right panel produces color changes by cutinase in the Zapek-Dox selective medium.

We investigated the effects of temperature, pH, metal ions, surfactants, and inhibitors on the activity of cutinase. The results showed that the recombinant proteins NsCut1-NsCut4 exhibited the highest activity at 40°C. Enzyme activity increased within the temperature range of 20-45°C, then gradually declined beyond 45°C, with almost complete inactivation observed at 70°C (**Figure 3A**). Regarding pH, enzyme activity increased within the range of 6.0-8.0, with pH 8.0 being optimal. Activity remained relatively stable within the pH range of 6.0-9.0 (**Figure 3B**). Among the metal ions, the inhibitory effects of Mn^2+^, Na^+^ and Cu^2+^ were significant at 5 mM, while the inhibitory effects of Mn^2+^ and Cu^2+^ were enhanced at 10 mM, and the promotional effects of Mg^2+^ and Fe^2+^ were obvious (**Figure 3C**). The surfactants Tween-80, SDS and EDTA significantly inhibited enzyme activity, with SDS being the most potent inhibitor (**Figure 3D**).

To confirm the cutinase activity of the purified recombinant proteins, we utilized the hyaline circle method and Zapek-Dox selective medium. The results showed that all four recombinant proteins formed clear zones on the glycerol tributyrate medium and exhibited color changes on the Zapek-Dox selective medium, indicating the presence of cutinase activity (**Figure 3E**). The enzyme activity reached 128.5-137.3 U mL^-1^ under optimal conditions (40°C, pH 8.0).

### 3.6 NsCut1-NsCut4 signaling peptide has secretory activity

**Figure 4:**
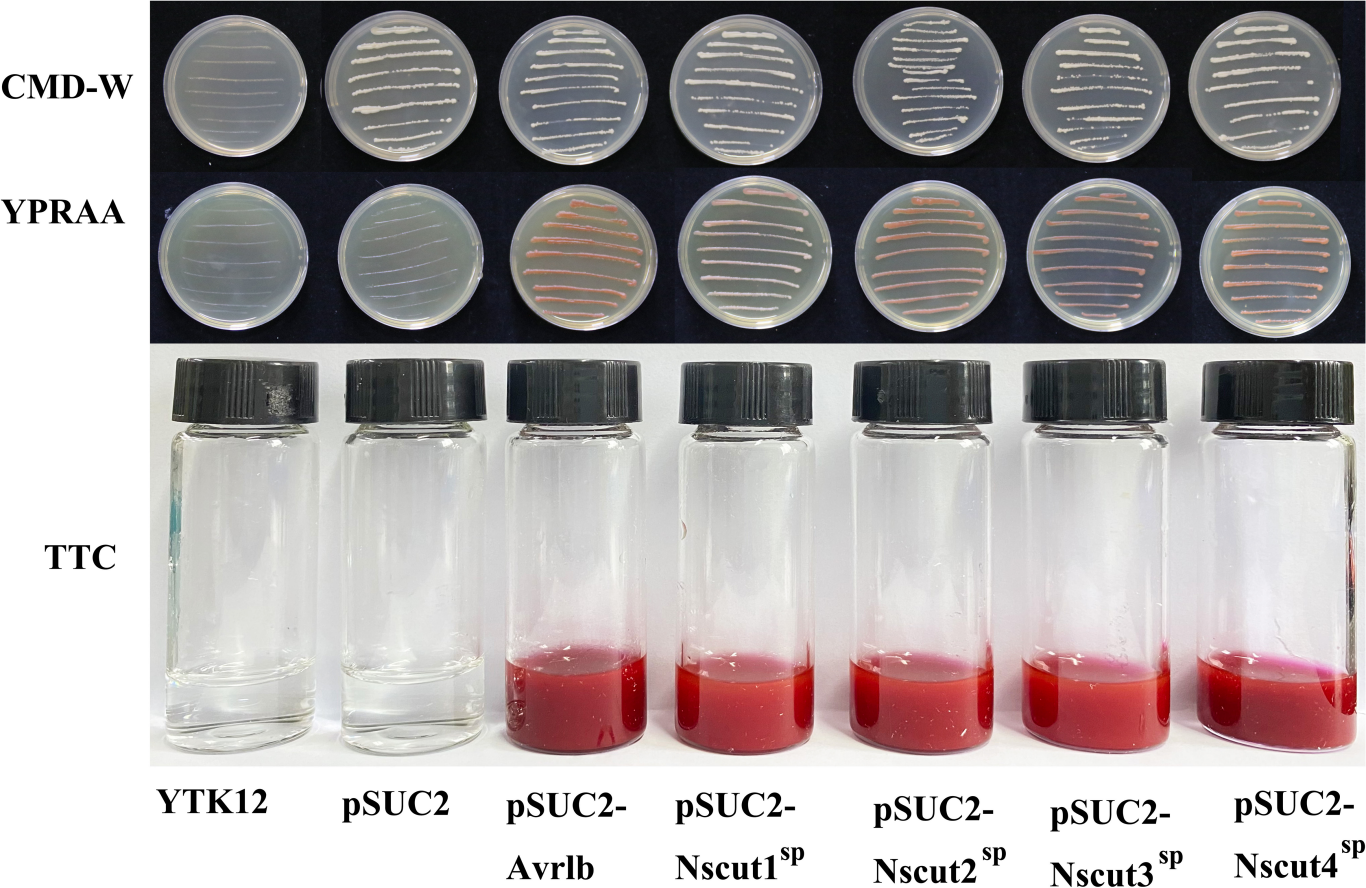
Validation of *NsCut1* to *NsCut4* protein signaling peptide secretion activity. Yeast strain YTK12 without vector, yeast strain YTK12 with vector pSUC2 (negative control), pSUC2-Avr1b^SP^ (positive control), and pSUC2-(NsCut1-NsCut4)^SP^ transformed yeast strain YTK12, were subjected to growth test and TTC color development assay on CMD_M and YPRAA agar medium.

The secretory function of the NsCut1-NsCut4 signaling peptide was validated using the Pichia yeast secretion system. The pSUC2-(NsCut1-NsCut4)^SP^ fusion vector was constructed and transformed into the defective yeast strain YTK12. The successful transformation was confirmed by gene amplification using the pSUC2-yz-F and pSUC2-yz-R primers (**Supplementary Figure 7**). The pSUC2-Avr1b^SP^ was used as a positive control, and the pSUC2 plasmid and empty vector YTK12 strain were used as negative controls. On CMD-W medium, the strains containing pSUC2, pSUC2-Avr1b^SP,^and pSUC2-(NsCut1-NsCut4)^SP^ grew normally, confirming the successful transformation of the vector (**Figure 4**). On YPRAA medium, both the positive control and the strain containing pSUC2-(NsCut1-NsCut4)^SP^ exhibited normal growth, while the negative control did not, thereby validating the secretion function of the target signal peptide (**Figure 4**). The TTC color development assay demonstrated that strains containing pSUC2-Avr1b^SP^ and pSUC2-(NsCut1-NsCut4)^SP^ were capable of reducing TTC to red TPF, whereas the negative control exhibited no color development (**Figure 4**). The results indicated that the NsCut1-NsCut4 signal peptide possesses secretory activity, consistent with the positive control.

### 3.7 NsCut1-NsCut4 proteins are all localized in the cell wall

**Figure 5:**
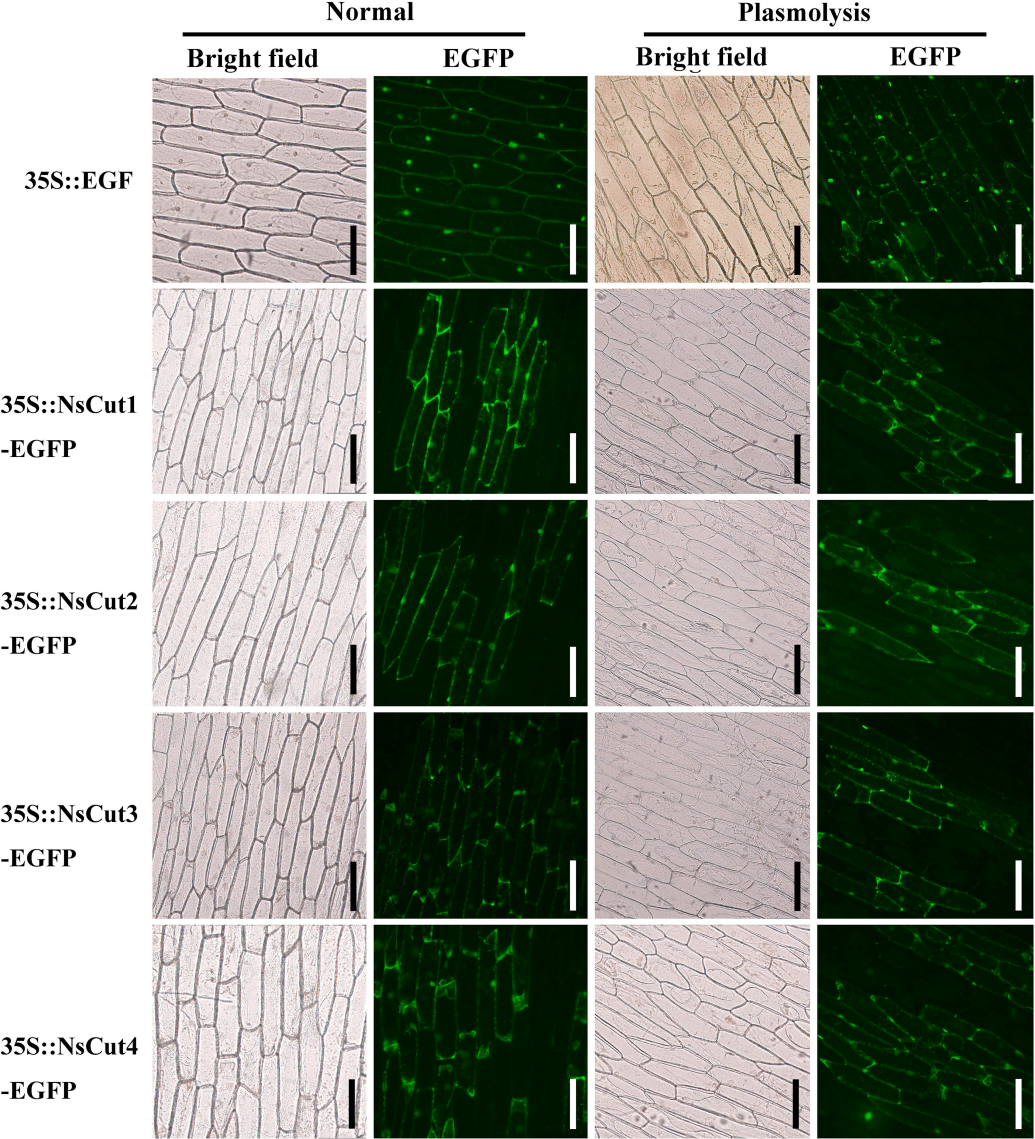
NsCut1-NsCut4 proteins are localized to the cell wall. Intact onion epidermal cells were observed under normal conditions in bright and fluorescent fields; 5 min after plasmodesmata separation, onion epidermal cell plasmodesmata were observed in bright and fluorescent fields with a scale bar of 200 µm.

The PCAMBIAsuper1300-GFP-(NsCut1-NsCut4) recombinant plasmid was transformed into *Agrobacterium tumefaciens* GV3101. Target bands were amplified using the 1300-yz-F and 1300-yz-R vector validation primers (**Supplementary Figure 8**). The recombinant plasmid GFP-(NsCut1-NsCut4) was introduced into onion epidermal cells via Agrobacterium-mediated method. Fluorescence confocal microscopy revealed that untransformed cells showed no fluorescence, while cells transformed with the GFP vector exhibited green fluorescence throughout the entire region. On the other hand, fluorescence in cells transformed with GFP-(NsCut1-NsCut4) was specifically localized to the cell membrane, with the fluorescence being predominantly concentrated in the cell wall after plasma wall separation (**Figure 5**). The results suggest that the NsCut1-NsCut4 protein is localized to the cell wall.

### 3.8 Successfully constructed knockout strains and complementary strains

The upstream and downstream homologous arms of the cutinase gene and the *hph* fragment were amplified using the genome of *Neostagonosporella sichuanensis* as a template. The knockout fusion vector was then constructed and verified to be correct through plasmid sequencing (**Supplementary Figures 9**, **10**). The fusion fragments were introduced into protoplasts via PEG-mediated transformation, and transformants were selected through Hygromycin B screening. Two rounds of PCR verification confirmed the successful knockout of the target genes NsCut1-NsCut4, with the positive transformants designated as *Δ*NsCut1-*Δ*NsCut4, respectively. Additionally, the complementation vector was constructed by incorporating the promoter and target gene sequences. Positive complementary strains, *Δ*NsCut1+-*Δ*NsCut4+, were obtained through screening following the introduction of the knockout mutants. Gene complementation was successfully verified by PCR (**Supplementary Figure 11**).

### 3.9 Δ*NsCut1* and Δ*NsCut3* showed significant changes in colony morphology, sporulation capacity and growth rate

**Figure 6:**
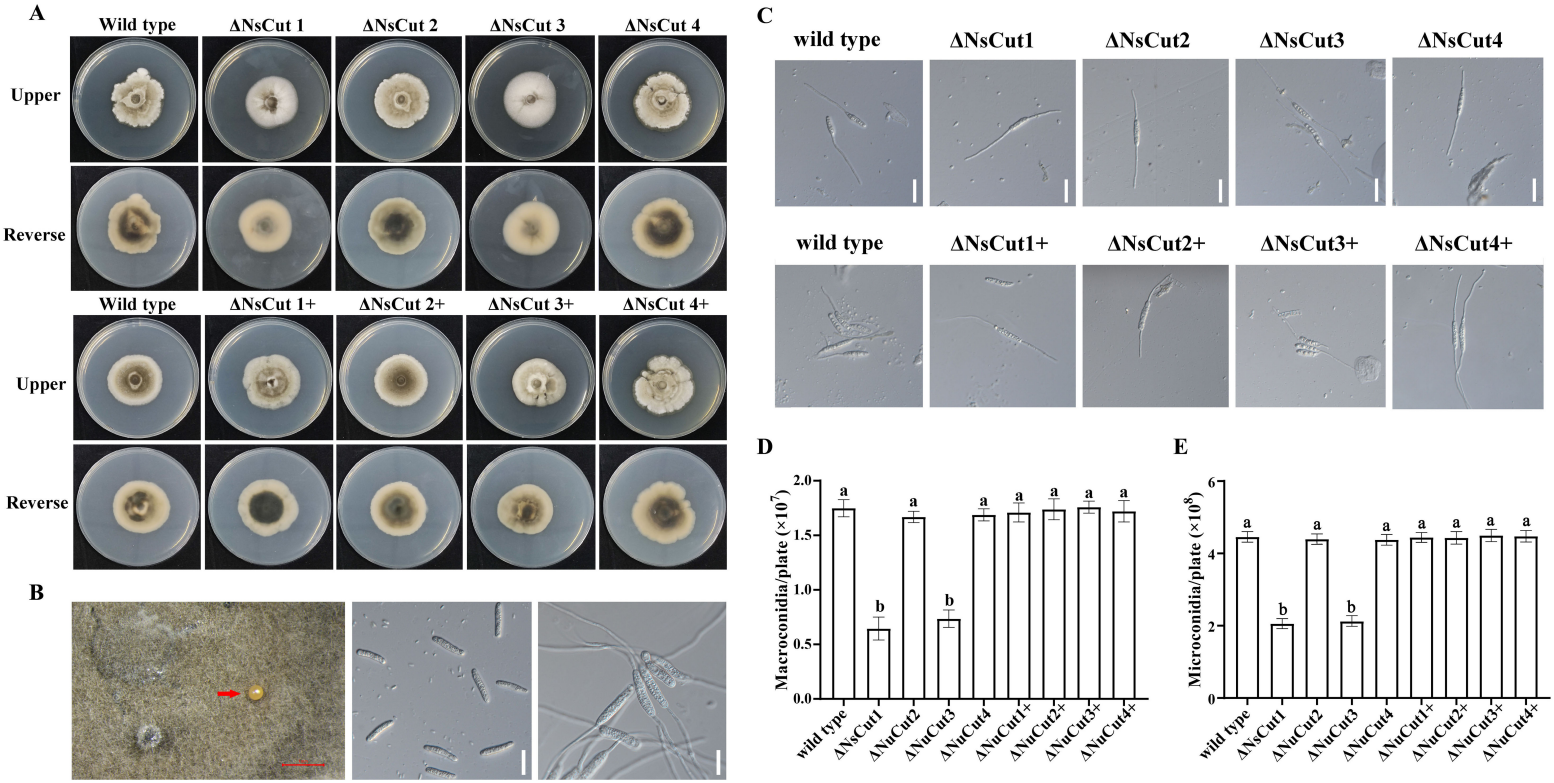
Colony morphology and spore germination of wild-type, knockout, and complementary strains. **(A)** Δ*NsCut* is the knockout strain and Δ*NsCut+* is the complementation strain. **(B)** Spore production in indoor cultures and germination of spores. The scale in the figure (red scale bar is 1 mm, white scale bar is 20 µm.) **(C)** Macroconidia germination of each strain, scale bar is 20 µm. **(D)** Statistics of macroconidia spore production of each strain. **(E)** Statistics of microconidia spore production of each strain. Different lowercase letters indicate significant differences in spore production of different strains under the same culture conditions (*P ≤* 0.05).

Wild-type, knockout, and complementary strains were inoculated on PDA and bamboo culm decoction agar medium to study colony morphology, growth rate, and spore production characteristics. On PDA plates, the *ΔNsCut1* and *ΔNsCut3* knockout strains exhibited light gray to white mycelium with a dense, fluffy texture and reduced pigmentation, whereas the *ΔNsCut2* and *ΔNsCut4* knockout strains displayed no significant morphological differences compared to the wild-type strain (**Figure 6A**). The morphology of the complementary strains *ΔNsCut1+* and *ΔNsCut3+* was restored to the wild-type level. No significant differences in growth rate were observed among the strains. On bamboo culm decoction agar medium, macroconidia of all strains germinated, while microconidia failed to germinate (**Figures 6B, C**). The *ΔNsCut1* and *ΔNsCut3* knockout strains exhibited significantly reduced spore production, which was restored to wild-type levels upon complementation, whereas the *ΔNsCut2* and *ΔNsCut4* knockout strains showed no significant difference in spore production compared to the wild-type strain (**Figures 6D, E**).

### 3.10 Knockouts had less effect on the overall enzyme activity of the strain

**Figure 7:**
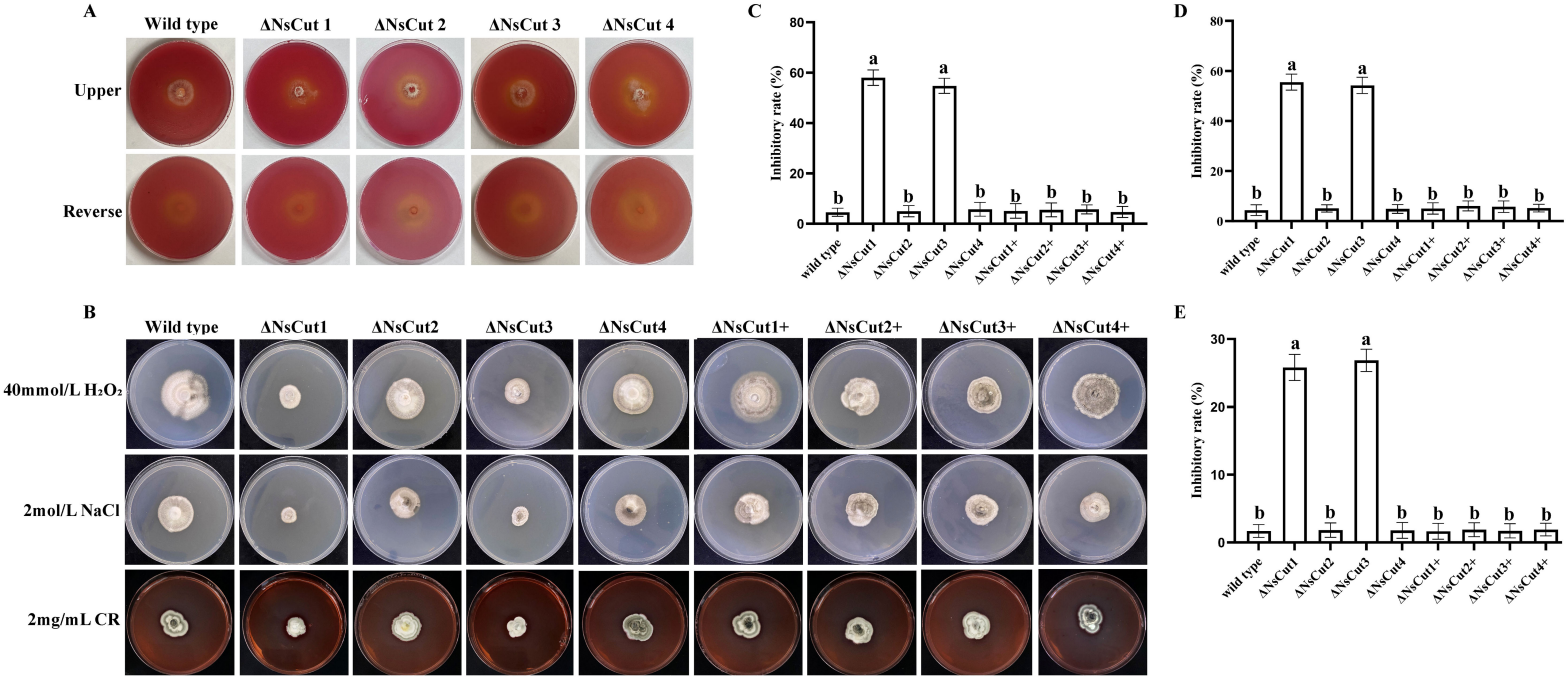
Enzymatic activity of each strain, colony morphology and growth inhibition under different stresses. **(A)** There was no significant difference in color change between wild-type and knockout strains on the Zapek-Dox medium. **(B)** The colony morphology of wild-type, knockout, and complementary strains under different stress treatments. **(C)** The growth inhibition of different strains under 40 mmol L^-1^ H_2_O_2_ stress. **(D)** The growth inhibition of different strains under 2 mol L^-1^ NaCl stress. **(E)** The growth inhibition of different strains under 2 mg mL^-1^ CR stress. Different lowercase letters indicate significant differences in the inhibition rates of different strains under the same stress treatment conditions (*P ≤* 0.05).

Wild-type and knockout strains were inoculated in Zapek-Dox medium and incubated at 25°C for 30 d, and cutinase activity was assessed by indicator color change (**Figure 7A**). The results indicated weak colony growth with sparse mycelium. No significant difference in indicator color change was observed, suggesting that the knockout of the cutinase gene had minimal impact on the overall enzymatic activity of the strains.

### 3.11 Δ*NsCut1* and Δ*NsCut3* were less stress tolerant than the wild-type strain

Knockout strains *ΔNsCut1* and *ΔNsCut3* exhibited significantly higher sensitivity to oxidative stress, osmotic stress, and cell wall stressors compared to the wild-type strain when cultured on PDA plates supplemented with stress-inducing factors (**Figure 7B**). Under H_2_O_2_, NaCl, and Congo red treatments, *ΔNsCut1* and *ΔNsCut3* showed a relative growth inhibition increase of 54.43%, 51.94%, and 24.12%, respectively, indicating reduced stress tolerance (**Figures 7C–E**). The tolerance of the complementary strains Δ*NsCut1+* and Δ*NsCut3+* was restored to the wild-type levels, suggesting that *NsCut1* and *NsCut3* play crucial roles in coping with oxidative stress, osmotic stress, and cell-wall inhibitor stress. In contrast, the stress response of Δ*NsCut2* and Δ*NsCut4* did not significantly differ from that of the wild-type strain.

### 3.12 Δ*NsCut1* and Δ*NsCut3* reduce strain virulence

**Figure 8:**
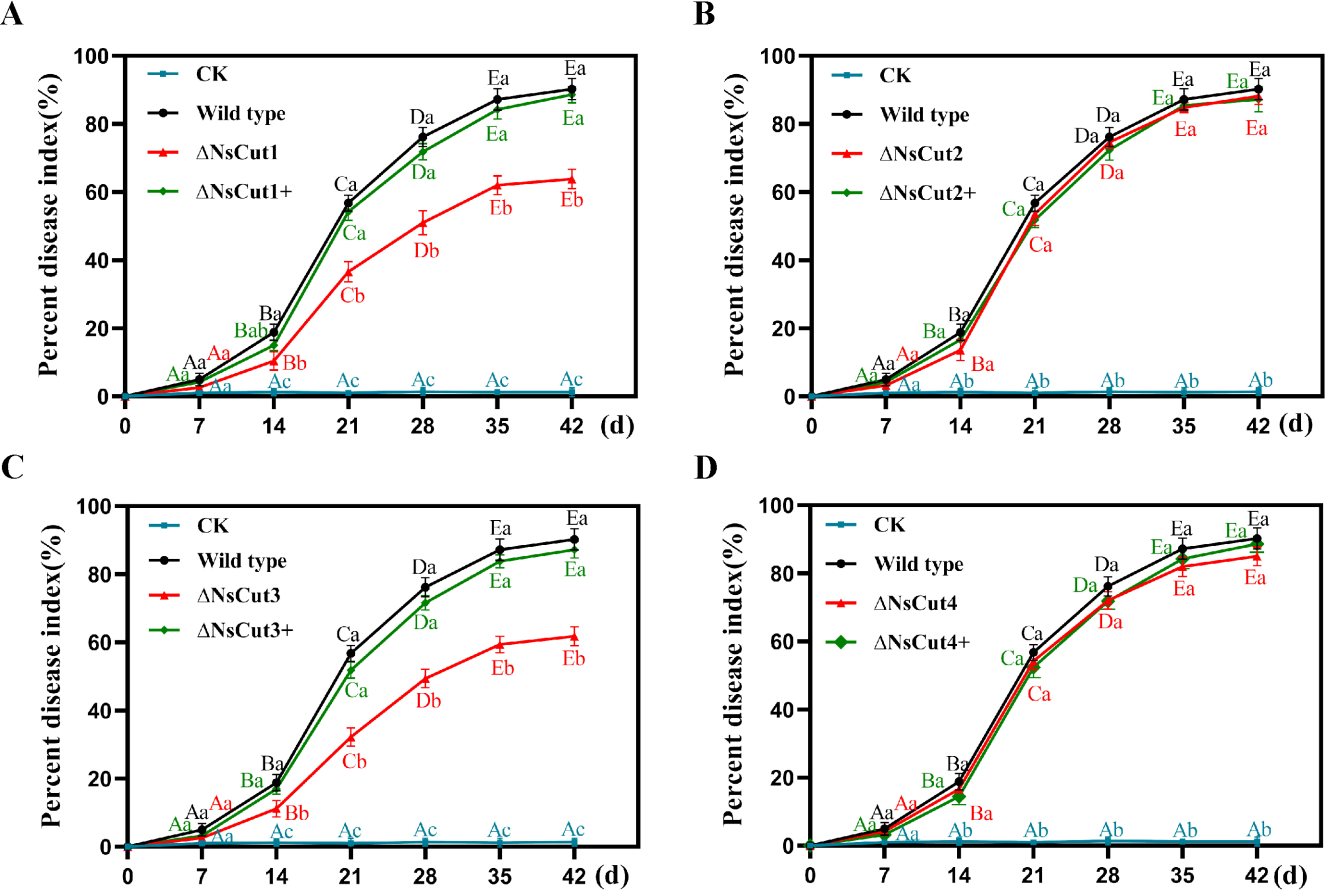
Pathogenicity analysis of wild-type, knockout, and complementary strains. **(A-D)** The changes in disease index of *Phyllostachys heteroclada* after inoculation with knockout and complementary strains of NsCut1-NsCut4, wild-type strains, and sterile water, respectively. Different lowercase letters indicate significant differences in the disease index of different strains at the same time, and different uppercase letters indicate significant differences in the disease index of the same strain at different times (*P ≤* 0.05).

In order to assess the effect of the four cutinase genes on the virulence of the strains, the disease index of different strains after inoculation was determined in this study (**Figure 8**). The results showed that the *ΔNsCut1* and *ΔNsCut3* knockout strains exhibited milder infection symptoms and a significantly lower disease index compared to the wild-type strain, whereas no significant difference in the disease index was observed between the wild-type and complementary strains (*ΔNsCut1+* and *ΔNsCut3+*) (**Figures 8A, C**). In contrast, the *ΔNsCut2* and *ΔNsCut4* knockout strains showed no significant change in disease index compared to the wild-type strain (**Figures 8B, D**).

## 4 Discussion

Cutinase is the most critical and essential tool for pathogens to break through the first barrier of host plants, which can help pathogens complete the processes of colonization, infestation, and reproduction (Chassot et al., 2007; Yan et al., 2022). Whether the cutinase gene of *Neostagonosporella sichuanensis* is involved in the infestation and colonization of the host, *Phyllostachys heteroclada*, is unknown. Therefore, this study focused on the structural characterization of the cutinase genes NsCut1-NsCut4 and their effects on cutinase activity and pathogenicity.

Bioinformatics analysis indicated that the amino acid sequences of the four cutinases all contained signal peptides at the N-terminus, without transmembrane structural domains, and that the four cutinase proteins contained at least one or more phosphorylated protein kinase sites, which is a structural feature in line with the properties of the cutinases that have been reported so far (Lin and Kolattukudy, 1980; Wang et al., 2023). In addition, the amino acid sequences of all three cutinases except *NsCut1* formed two pairs of disulfide bonds, whereas *NsCut1* could form four pairs of disulfide bonds. These disulfide bonds helped to maintain the spatial structure of the cutinases stable (Wang et al., 2020a). Most cutinases contain a conserved GYSQG structural domain, which plays a vital role in the function and stability of cutinases (Longhi et al., 1997; Chen et al., 2013). The *NsCut1*, *NsCut2*, and *NsCut3* protein sequences have a conserved GYSQG catalytic site. However, the catalytic site of *NsCut4* protein has been replaced by “GFSQG” instead of “GYSQG.” The specific function of this mutation has yet to be investigated. By comparing the amino acid sequences of the four cutinases with those of five fungal cutinases under Phaeosphaeriaceae, we found that the different cutinases of the same species were not clustered together. This result suggests that the evolutionary relationships of cutinases show diversity in Phaeosphaeriaceae, and this diversity may originate from the important role of cutinases in the growth and reproduction process of fungi, which has led to gradual differences in the evolutionary process (Arya and Cohen, 2022).

We successfully obtained four cutinase gene deletion mutants by PEG-mediated knockdown of protoplasts and excluded epigenetic changes caused by mutagenicity of protoplast transformation itself using knockdown complementary assay (You et al., 2009). It was observed that Δ*NsCut1* and Δ*NsCut3* knockout transformants resulted in lighter colony color and reduced spore production. At the same time, the complementary strains showed no significant difference in colony morphology and spore production from the wild type. For the lighter colony color, it might suggest that these two genes transport fungal pigments or precursors for melanin synthesis or are directly involved in melanin synthesis. DOPA and DHN are the most studied melanins in phytopathogenic fungi and they are closely related to the virulence and pathogenicity of pathogenic fungi (Cao et al., 2006; Zhou et al., 2011; Chen et al., 2017). Melanins are required for the invasion of hosts by many fungi such as *Colletotrichum orbiculare*, *Colletotrichum lindemuthianum* and *Colletotrichum graminicola* (Wolkow et al., 1983; Rasmussen and Hanau, 1989; Perpetua et al., 1996). The decrease in spore production is presumed to be related to the transport of certain nutrients, leading to a decrease in the utilization of nutrients by the fungus, which in turn affects the decrease in spore production. In contrast, the knockout strains, Δ*NsCut2* and Δ*NsCut4*, did not differ significantly from the wild-type strain regarding colony morphology characteristics, growth rate, and spore production process.

In *N. sichuanensis*, several genes can degrade *P. heteroclada* epidermal tissues and cell walls in addition to cutinase genes. Therefore, we found little difference in color change between wild-type and knockout strains of *N. sichuanensis* on the Zapek-Dox cutinase screening medium. Similarly, the cutinase enzyme activity of the *SsCut1* knockout transformants of the cutinase gene of *Sclerotinia sclerotiorum* did not differ significantly from that of the wild-type strain (Gong et al., 2022). It suggests that in the absence of the cutinase gene, other genes may play complementary roles, thus making the cutinase enzyme activity of the knockout strain comparable to that of the wild-type strain.

During plant-pathogen interactions, the reactive oxygen species (ROS) burst of the host plant is an essential line of defense against pathogen invasion, and the ability of the pathogen to cope with reactive oxygen species is critical to its ability to break through the host’s defenses (Torres and Dangl, 2005; Torres et al., 2005; Gan et al., 2009; Zhang et al., 2009; L’Haridon et al., 2011). Δ*NsCut1* and Δ*NsCut3* knockout strains were significantly more sensitive than the wild-type strains to a wide range of stresses, suggesting that Δ*NsCut1* and Δ*NsCut3* enhance the pathogen sensitivity to extracellular oxidative stress, suggesting that they play a crucial role in ROS detoxification and suppress plant immune responses. This finding is consistent with the fact that loss of function of the *ApCtf1β1* and *ApCtf1β2* genes of *Arthrinium Phaeospermum* results in enhanced susceptibility of the pathogen to extracellular oxidative stress (Fang et al., 2021). In addition, the loss of function of *NsCut1* and *NsCut3* genes resulted in increased sensitivity of *N. sichuanensis* cell wall to external disturbance stress, which coincided with the changes in stress sensitivity caused by the deletion of *BbCtflα* and *BbCtflβ* genes in *Beauveria bassiana* (Wang et al., 2020b). The critical role of *NsCut1* and *NsCut3* genes in maintaining cell stability was further confirmed.

Cutinases not only activate the plant’s immune response but also penetrate the outermost cuticle barrier of the host to invade the plant (Silva et al., 2010; Ortiz-Urquiza and Keyhani, 2013). In other plant pathogens, the deletion of genes encoding cutinases has been shown to reduce virulence or eliminate pathogenicity in the host plant (Zhang et al., 2005; Liu et al., 2016; Wang et al., 2016; Gui et al., 2017). In this study, Δ*NsCut1* and Δ*NsCut3* knockout strains infected *P. heteroclada* with significantly less damage than wild-type strains infested with them, whereas Δ*NsCut2* and Δ*NsCut4* deletion mutants did not differ significantly from the wild-type in terms of pathogenicity. Thus, knockout strains lacking the *NsCut1* and *NsCut3* genes are hampered in attacking the first barrier of the host plant, as they are incapable of producing and releasing the enzymatic tools for degrading *P. heteroclada* cuticle, making it difficult for the pathogen to invade and colonize the host plant cells. Knockout of the gene encoding cutinase has been reported to have no significant effect on the virulence of specific plant pathogenic fungi due to the functional redundancy of multiple genes encoding cutinase in the genome (Stahl and Schfer, 1992; Crowhurst et al., 1997; Chen et al., 2021). Therefore, even if the deletion of the cutinase gene results in the blockage of the initial stage of invasion, the processes of colonization, infestation, and reproduction in *P. heteroclada* cells are no longer constrained once *N. sichuanensis* has successfully invaded the host plant through other routes. That is why it is equally crucial for us to study the function of cutinase genes and related genes involved in colonization and infestation during the interaction between the pathogen and the host. This helps us to find practical breakthroughs to prevent and control fishscale bamboo rhombic spots from the development of the disease process and the synergistic evolution of the pathogen and the host.

## Data Availability

The datasets presented in this study can be found in online repositories. The names of the repository/repositories and accession number(s) can be found in the article/**Supplementary Material**.
