# Supplementary material for: Functional characterization of cutinase genes NsCut1-NsCut4 in Neostagonosporella sichuanensis and their effects on fishscale bamboo
Source: Front Plant Sci. 2025 Apr 8;16:1564651. doi: 10.3389/fpls.2025.1564651 (PMC12011716; doi:10.3389/fpls.2025.1564651)
Supplement: Supplementary file 1 [file DataSheet1.pdf]

Supplementary Material

1 Supplementary Tables

Supplementary Table 1. Sequence list of primers for gene cloning

Primers	Primer sequences (5′–3′)
NsCut1-F	ATGAAGGTCACCGCAGCAG
NsCut1-R	CTATGCAGTGAGCTTGCTCTTGA
NsCut2-F	ATGTACACACAAACAGCTCTCCTTT
NsCut2-R	TCATGCCACGAGCCCCT
NsCut3-F	ATGCACTTCTCCACACCCA
NsCut3-R	CTAAGCACCTAAGGACGTCTCA
NsCut4-F	ATGTCTTCCATCATCCGCAA
NsCut4-R	TTAAACGTAGACGTACTCAATGTCC

Supplementary Table 2. Sequence list of primers for prokaryotic expression and protein purification

Primers	Primer sequences (5′–3′)
NsCut1-*EcoR*I-F	caggggcccggatccgaattcGGCCCAGTCGAGATTGCC
NsCut1-*Xho*I-R	gtggtggtggtggtgctcgagCTATGCAGTGAGCTTGCTCTTGA
NsCut2-*EcoR*I-F	caggggcccggatccgaattcAGCCCTCTCGCGGTTCGC
NsCut2-*XhoI*-R	gtggtggtggtggtgctcgagTCATGCCACGAGCCCCTC
NsCut3-*EcoR*I-F	caggggcccggatccgaattcGCTCCAATCGAAGAGCGCG
NsCut3-*Xho*I-R	gtggtggtggtggtgctcgagCTACATCAGCAAACGATCTTTAACCC
NsCut4-*EcoR*I-F	caggggcccggatccgaattcCAGGCCCCCAAGCTCGAC
NsCut4-*Xho*I-R	gtggtggtggtggtgctcgagTTAAACGTAGACGTACTCAATGTCCA
T7-F	TAATACGACTCACTATAGG
T7-R	GCTAGTTATTGCTCAGCG

*The restriction sites for vector construction are underlined; lower case letters are homologous arm sequences.

Supplementary Table 3. Sequence list of primers used to construct signal peptide secretion validation vectors

Primers	Primer sequences (5′–3′)
pSUC2-yz-F	ATGACAAAAAAATGATGGAAGAC
pSUC2-yz-R	TGTGAAGAAGTGGACCAAAGG
pSUC2-NsCut1^{sp} -F	cggaattttaattaagaattcATGAAGGTCACCGCAGCAGC
pSUC2-NsCut1^{sp} -R	cactatagggagaacctcgagGCCGGCGGCGGCGAGGCC
pSUC2-NsCut2^{sp} -F	cggaattttaattaagaattcATGTACACACAAACAGCTCTCCTTTC
pSUC2-NsCut2^{sp} -R	cactatagggagaacctcgagGGCTGTGGCGAAGGCGGC
pSUC2-NsCut3^{sp} -F	cggaattttaattaagaattcATGCACTTCTCCACACCCATATC
pSUC2-NsCut3^{sp} -R	cactatagggagaacctcgagTGCTGTGGAGAGGGCAGCG
pSUC2-NsCut4^{sp} -F	cggaattttaattaagaattcATGTCTTCCATCATCCGCAACG
pSUC2-NsCut4^{sp} -R	cactatagggagaacctcgagAGCGGCAGCGACGGCGAC

*The restriction sites for vector construction are underlined; lower case letters are homologous arm sequences.

Supplementary Table 4. Sequence list of primers used to construct subcellular localization vectors

Primers	Primer sequences (5′–3′)
GFP-yz-F	GACGCCATTTCGCCTTTTC
GFP-yz-R	GCCGTCCAGCTCGACCAG
GFP-NsCut1-F	atacaccaaatcgactctagaATGAAGGTCACCGCAGCAGC
GFP-NsCut1-R	gcccttgctcaccatggtaccCTATGCAGTGAGCTTGCTCTTGA
GFP-NsCut2-F	atacaccaaatcgactctagaATGTACACACAAACAGCTCTCCTTTC
GFP-NsCut2-R	gcccttgctcaccatggtaccTGCCACGAGCCCCTCCAC
GFP-NsCut3-F	atacaccaaatcgactctagaATGCACTTCTCCACACCCATATC
GFP-NsCut3-R	gcccttgctcaccatggtaccCATCAGCAAACGATCTTTAACCC
GFP-NsCut4-F	atacaccaaatcgactctagaATGTCTTCCATCATCCGCAACG
GFP-NsCut4-R	gcccttgctcaccatggtaccAACGTAGACGTACTCAATGTCCATG

*The restriction sites for vector construction are underlined; lower case letters are homologous arm sequences.

Supplementary Table 5. Sequence list of primers used to construct knockout vectors

Primers	Primer sequences (5′–3′)
NsCut1-A1-F1	ttcggatcttccagagatatcATGGCTTCAAGATTCCCACCG
NsCut1-A1-R1	cttctgtcgacTTATTTGTGTCATTTGTGAGCGG
NsCut1-hph-F1	cacaaataaGTCGACAGAAGATGATATTGAAGGAG
NsCut1-hph-R1	TTACTATTCCTTTGCCCTCGGA
NsCut1-A2-F1	cgagggcaaaggaatagtaaATGTCTCTGACTACAGTGAAAACTTGC
NsCut1-A2-R1	caactgccgttcgacgatatcTTAATAAGACGAGCGGCTGGG
NsCut2-A1-F1	ttcggatcttccagagatatcATGTGAAGCACAGGAGATTTTCTTT
NsCut2-A1-R1	tcttctgtcgacTCAGTGCTGCCAATTCCGC
NsCut2-hph-F1	agcactgaGTCGACAGAAGATGATATTGAAGGAG
NsCut2-hph-R1	TTACTATTCCTTTGCCCTCGGA
NsCut2-A2-F1	cgagggcaaaggaatagtaaATGCCAATGCTTGTTAAAATGTTG
NsCut2-A2-R1	caactgccgttcgacgatatcTCAGACGTTGGGGAATCGTT
NsCut3-A1-F1	ttcggatcttccagagatatcATGACTACTTGGACGATATCGACTCT
NsCut3-A1-R1	cttctgtcgacCTAATCTAGTACTTTTAGTCTATCCAAAGTCG
NsCut3-hph-F1	ctagattagGTCGACAGAAGATGATATTGAAGGAG
NsCut3-hph-R1	TTACTATTCCTTTGCCCTCGGA
NsCut3-A2-F1	cgagggcaaaggaatagtaaATGCTAAACGACTATATTTGTATTGTGAA
NsCut3-A2-R1	caactgccgttcgacgatatcTCATGCGACTTTCTTTGCGG
NsCut4-A1-F1	ttcggatcttccagagatatcATGCTGTCGATCGTTTGGTTCT
NsCut4-A1-R1	cTCATAATGCCTTTTCGGCGG
NsCut4-hph-F1	cgccgaaaaggcattatgaGTCGACAGAAGATGATATTGAAGGAG
NsCut4-hph-R1	cgacggtgatgcttttccatTTACTATTCCTTTGCCCTCGGA
NsCut4-A2-F1	ATGGAAAAGCATCACCGTCG
NsCut4-A2-R1	caactgccgttcgacgatatcTAATCCCGGCGAGCTGTACC

* The restriction sites for vector construction are underlined; lower case letters are homologous arm sequences.

Supplementary Table 6. List of primer sequences used to verify successful knockout vector construction

Primers	Primer sequences (5′–3′)
NsCut1-A1-F2	CTCGCTGCTTGCTATCTC
NsCut1-hph-R2	CACGCCATGTAGTGTATTGA
NsCut1-hph-F2	TCCTGCGGGTAAATAGCT
NsCut1-A2-R2	GATTGGAACAAGGTCGTG
NsCut2-A1-F2	GAATAGCCAGGATCACAAC
NsCut2-hph-R2	AAAGTGCCGATAAACATAAC
NsCut2-hph-F2	TGTCCTGCGGGTAAATAG
NsCut2-A2-R2	TAGATCCAGCGTCATCGT
NsCut3-A1-F2	TCTGAGTGGTGCATTGGC
NsCut3-hph-R2	TGTTGGCGACCTCGTATT
NsCut3-hph-F2	TGTCCTGCGGGTAAATAG
NsCut3-A2-R2	TCTGTGCTGCGTTCTGTG
NsCut4-A1-F2	GCAGTTTATGCCATTTACG
NsCut4-hph-R2	GCTATTTACCCGCAGGAC
NsCut4-hph-F2	GCAAGACCTGCCTGAAAC
NsCut4-A2-R2	GACGCCGAGACCGTAAAG

2 Supplementary Figures

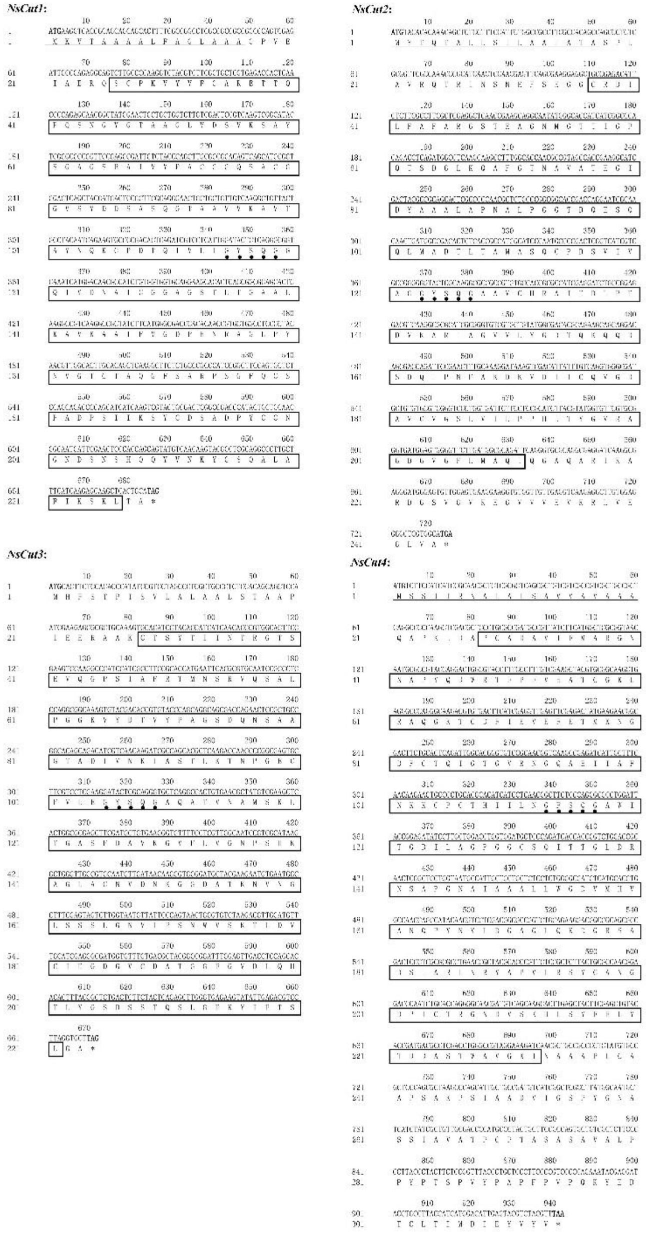

Supplementary Figure 1. Sequence of the cDNA of the cutinase genes of NsCut1-NsCut4 from *N. sichuanensis* and the amino acid sequences encoded by them

Note: ATG marked by double solid lines is the start codon, *is the stop codon; Amino acids marked solid line are signal peptides; dots are GYSQG catalytic sites; the Cutinase domain are indicated by boxes.

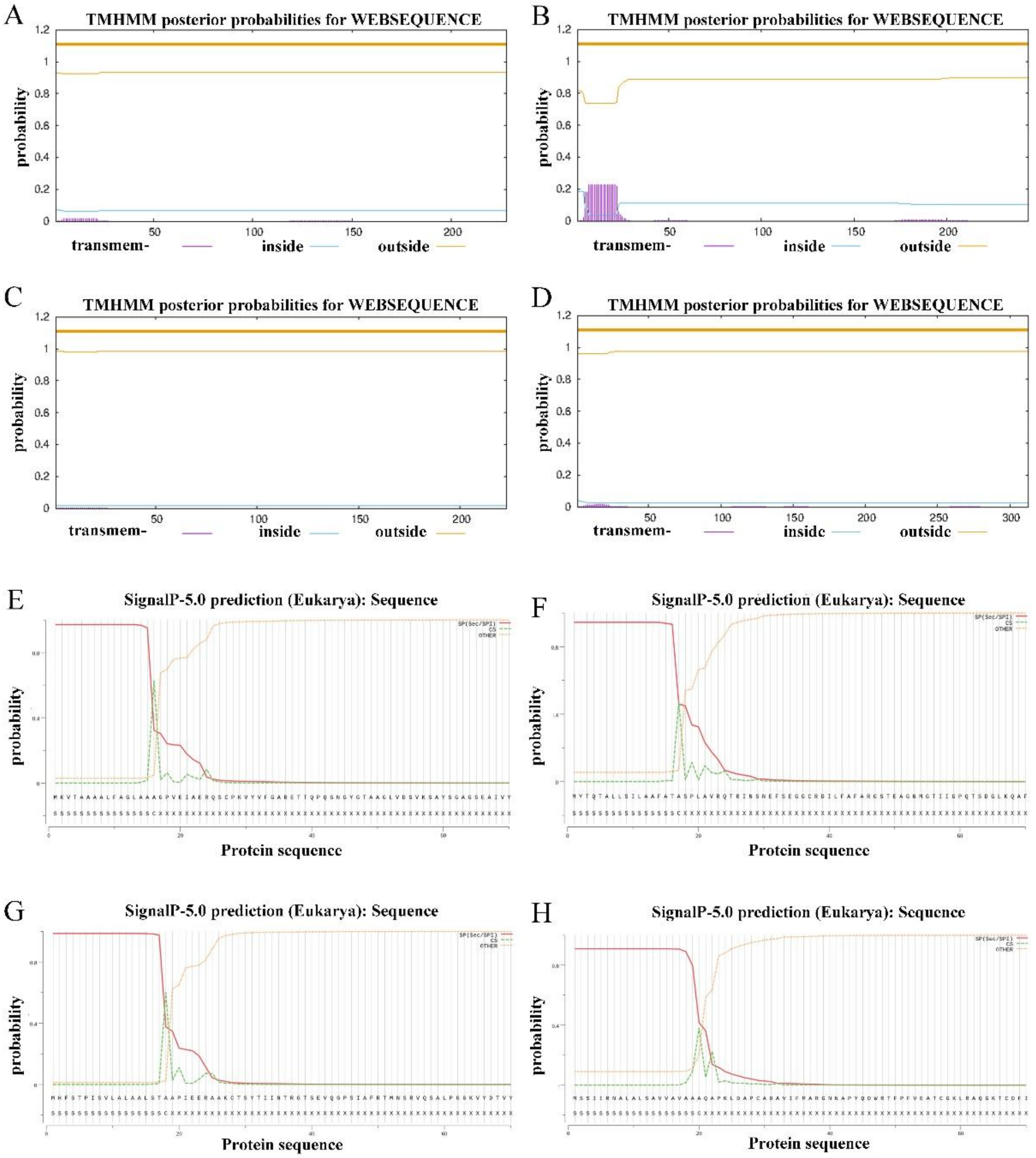

Supplementary Figure 2. Protein structure prediction of *NsCut1* to *NsCut4*

Note: (A-D) Prediction of protein transmembrane domains. (E-H) Prediction of protein signal peptide.

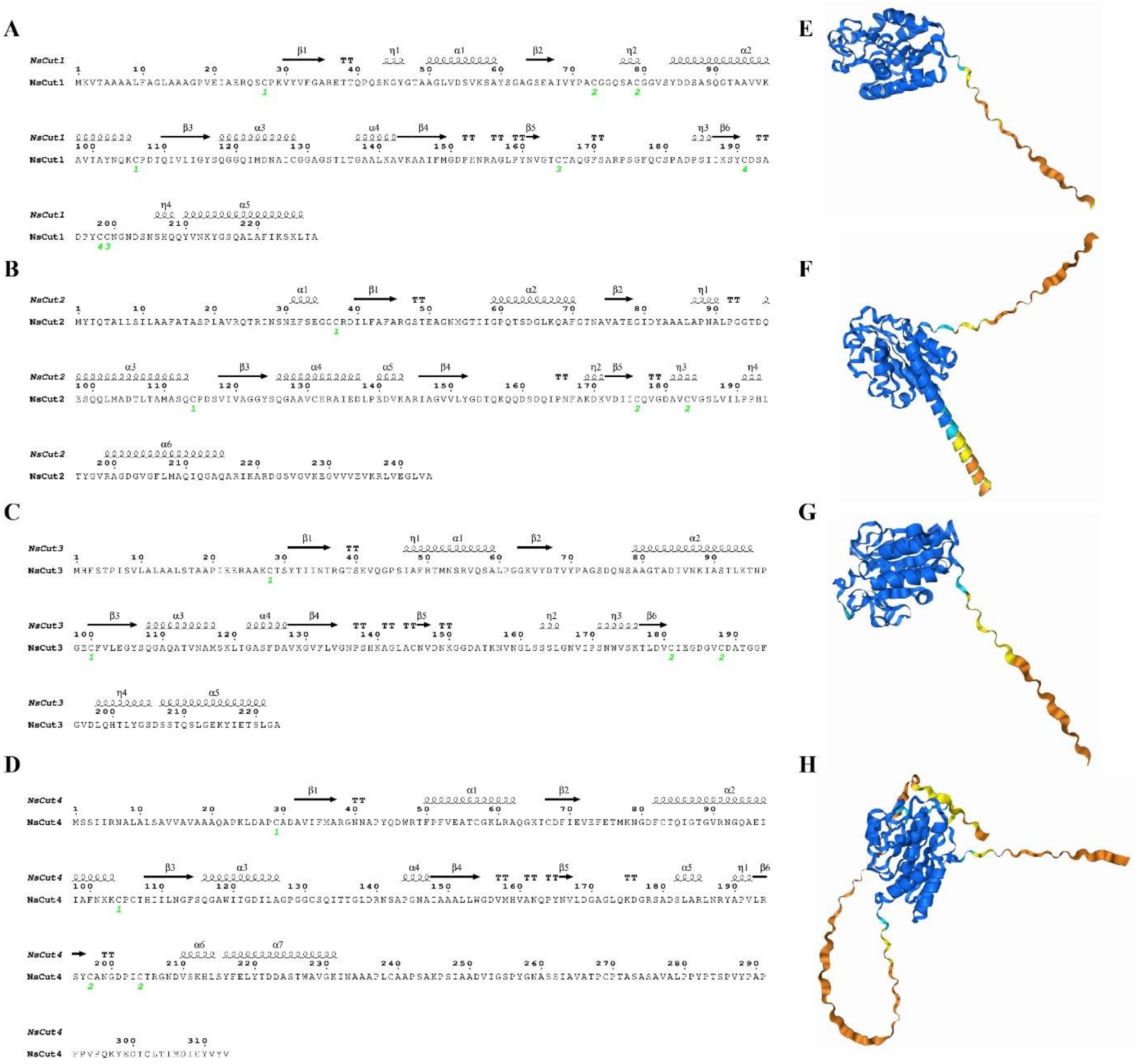

Supplementary Figure 3. Secondary and tertiary structure of *NsCut1* to *NsCut4*

Note: (A-D) are the secondary structures of NsCut1-NsCut4. The α-helix, η-helix, β-fold and TTT are indicated. green numbers represent the positions of disulfide bonds; (E-H) are the tertiary structure of NsCut1-NsCut4. More blue parts indicate a higher confidence level.

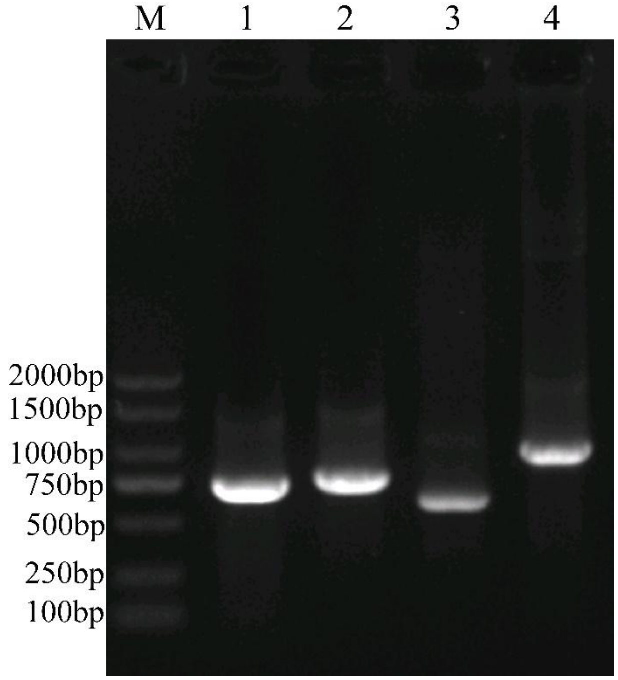

Supplementary Figure 4. Electrophoretogram of target fragment clones

Note: M: DL2000 DNA marker; 1: *NsCut1* target segment; 2: *NsCut2* target segment; 3: *NsCut3* target segment; 4: *NsCut4* target segment.

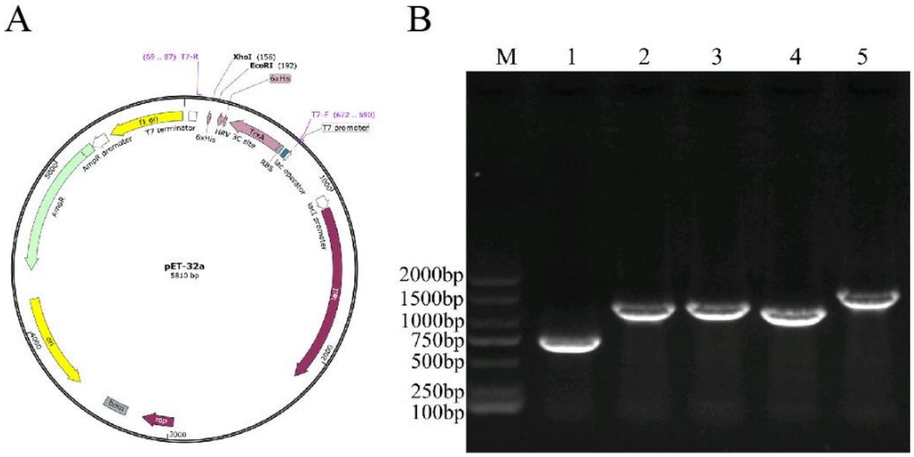

Supplementary Figure 5. Plasmid map of pET-32a and the results for identification of positive colonies

Note: (A) Plasmid map of pET32a; (B) M: DL2000 DNA marker; lane 1: PCR amplification product of the empty pET32a vector; lane 2: PCR amplification product of the pET32a-*NsCut1* recombinant plasmids; lane 3: PCR amplification product of the pET32a-*NsCut2* recombinant plasmids; lane 4: PCR amplification product of the pET32a-*NsCut3* recombinant plasmids; lane 5: PCR amplification product of the pET32a-*NsCut4* recombinant plasmids.

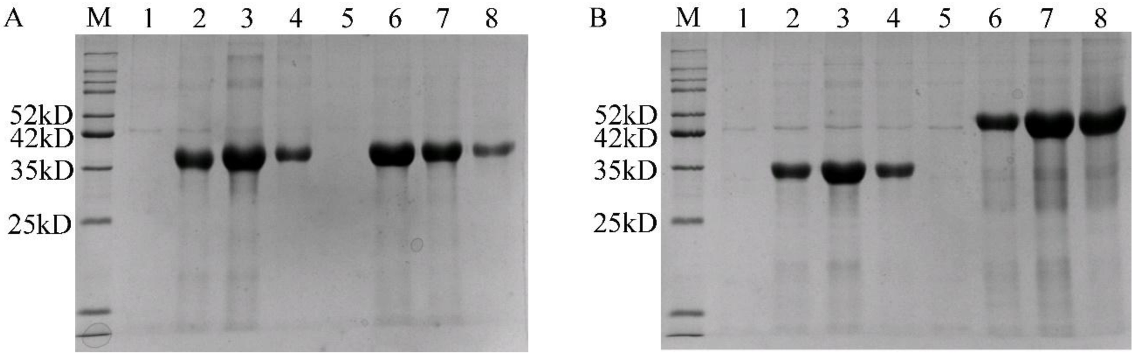

Supplementary Figure 6. Analysis of the recombinant *NsCut1* to *NsCut4* protein with SDS-PAGE

Note: M: Protein Marker. (A) 1-4 are recombinant protein *NsCut1* washed last 1 time and eluted 1 to 3 times, 5-8 are recombinant protein *NsCut2* washed last 1 time and eluted 1 to 3 times. (B) 1-4 for recombinant protein *NsCut3* washed last 1 time and eluted 1 to 3 times, 5-8 for recombinant protein *NsCut4* washed last 1 time and eluted 1 to 3 times.

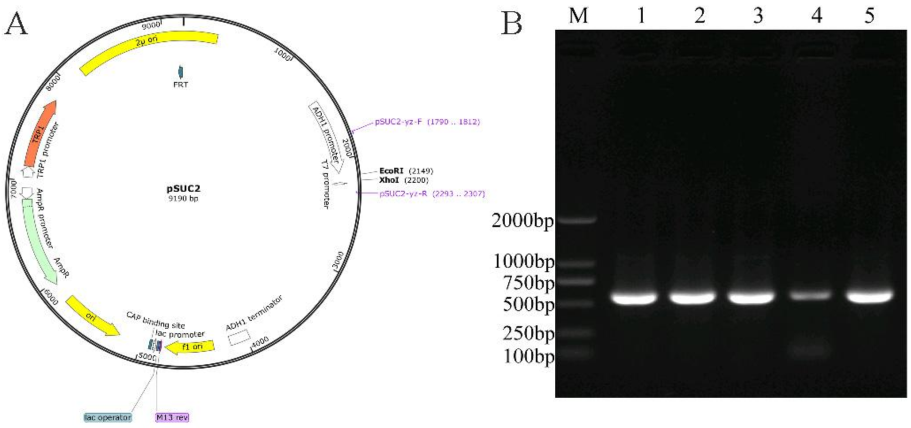

Supplementary Figure 7. Plasmid map of pSUC2 and the results for identification of positive colonies

Note: (A) Plasmid map of pSUC2; (B) M: DL2000 DNA marker; lane 1-5: PCR amplification product of the empty pSUC2 vector, pSUC2-*NsCut1*^{SP}, pSUC2-*NsCut2*^{SP}, pSUC2-*NsCut3*^{SP} and pSUC2-*NsCut4*^{SP}.

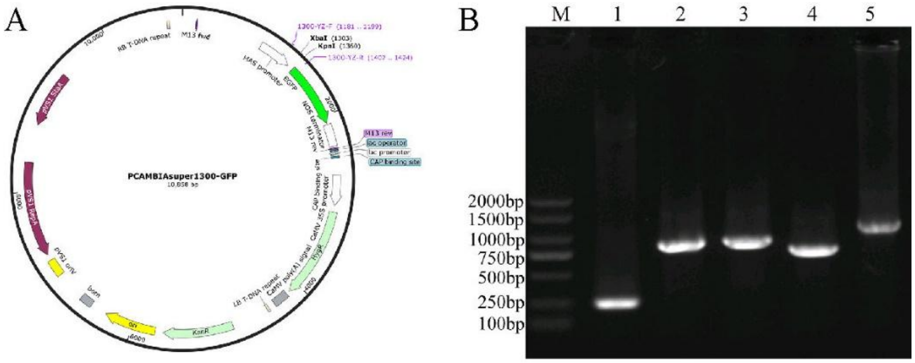

Supplementary Figure 8. Plasmid map of PCAMBIAsuper1300-GFP and the results for identification of positive colonies

Note: (A) Plasmid map of PCAMBIAsuper1300-GFP; (B) M: DL2000 DNA marker; lane 1-5: PCR amplification product of the empty GFP vector, GFP-*NsCut1*, GFP-*NsCut2*, GFP-*NsCut3* and GFP-*NsCut4* recombinant plasmids.

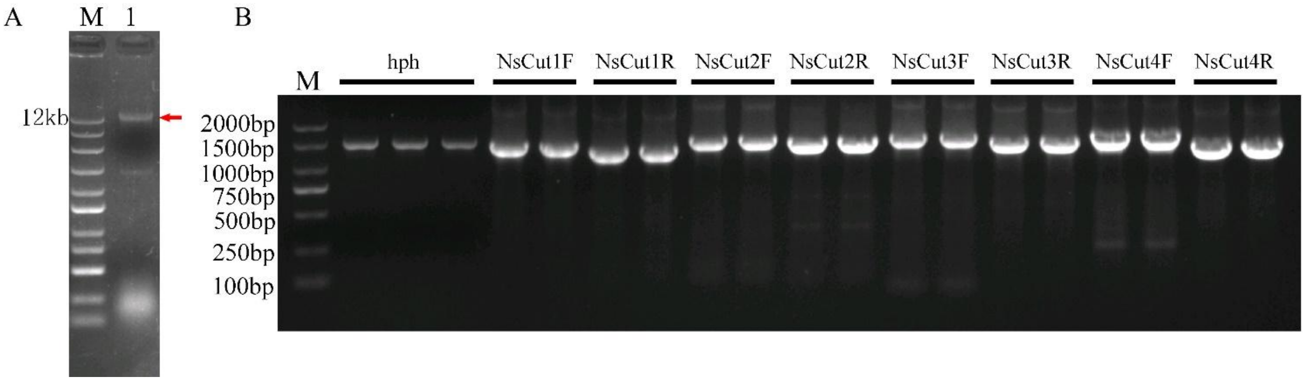

Supplementary Figure 9. Construction of NsCut1-NsCut4 knockout frames

Note: (A) Extraction of genomic DNA from *N. sichuanensis*. (B) Electrophoretic detection of homologous arms of target genes and *hph* fragments. M: DL2000 DNA marker.

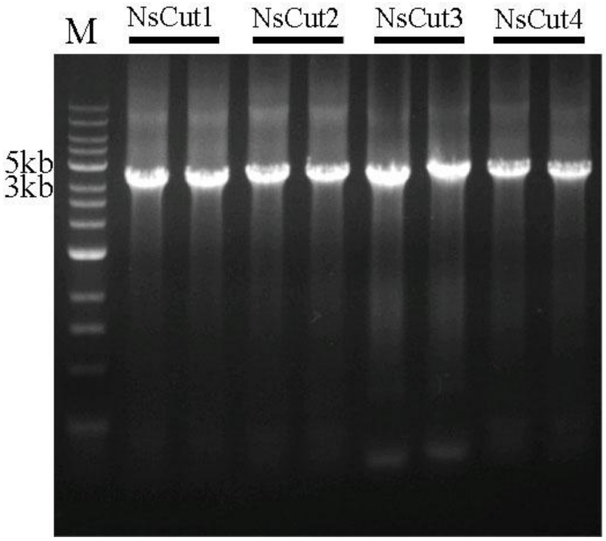

Supplementary Figure 10. Electrophoretic detection of fusion fragments

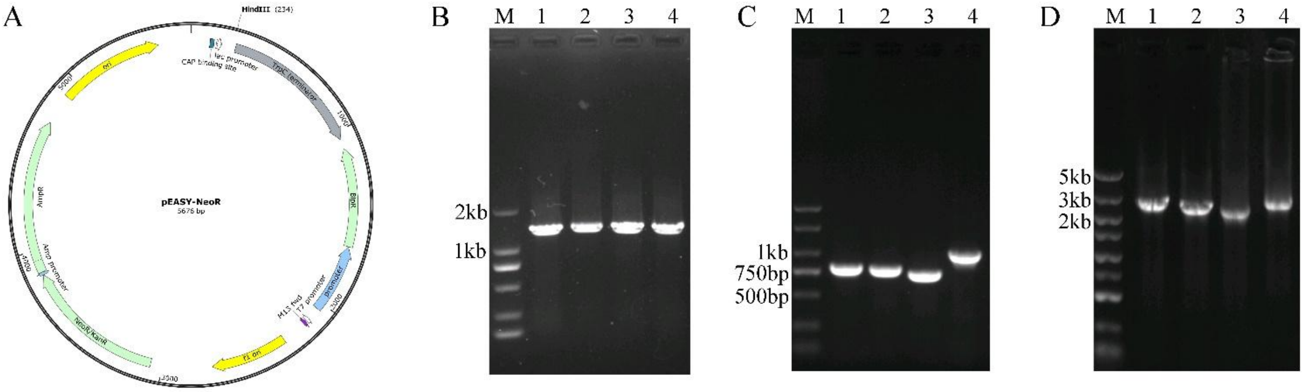

Supplementary Figure 11. Plasmid map of pEASY-NeoR plasmid mapping, promoter fragments, target genes and complementary sequence fusion fragments

Note: (A) Plasmid map of pEASY-NeoR; (B) Promoter fragment of NsCut1-NsCut4; (C) Target genes of NsCut1-NsCut4; (D) Complementary sequence fusion fragments of NsCut1-NsCut4.
